# Enantioselective [3 + 2] annulation of α-substituted allenoates with β,γ-unsaturated *N*-sulfonylimines catalyzed by a bifunctional dipeptide phosphine

**DOI:** 10.3762/bjoc.12.37

**Published:** 2016-02-24

**Authors:** Huanzhen Ni, Weijun Yao, Yixin Lu

**Affiliations:** 1 Department of Chemistry, National University of Singapore, 3 Science Drive 3, Singapore, 117543

**Keywords:** [3 + 2] annulation, α-substituted allenoate, dipeptide phosphine, enantioselective

## Abstract

The first enantioselective [3 + 2] annulation of α-substituted allenoates with β,γ-unsaturated *N*-sulfonylimines is described. In the presence of a dipeptide phosphine catalyst, a wide range of highly functionalized cyclopentenes bearing an all-carbon quaternary center were obtained in moderate to good yields and with good to excellent enantioselectivities.

## Introduction

**Scheme 1:**
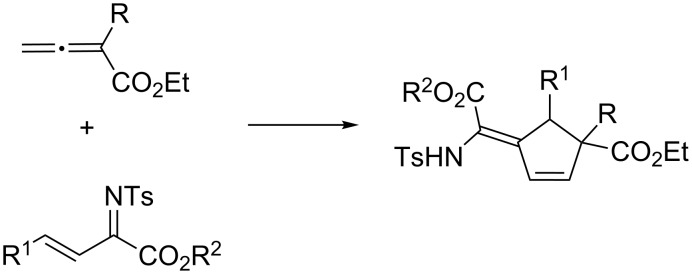
The [3 + 2] annulation of α-substituted allenoates reported by He.

Over the past decade, chiral phosphine catalysts have been utilized extensively for the construction of a broad range of synthetically useful molecular structures [1–13]. Since the initial discovery of phosphine-catalyzed [3 + 2] annulation of allenoates and activated alkenes by Lu in 1995, this type of annulation reaction has received considerable attention due to its high efficiency and versatility in creating five-membered ring systems [14–33]. However, most of the earlier examples make use of allenoates without an α-substitution. As demonstrated by Yu, Kwon and their co-workers [34–36], this is due to the requirement of a hydrogen atom at the α-position for a proton shift during the reaction cycle. Instead, α-substituted allenoates were shown to interact with phosphine in different reaction modes and undergo [4 + 2] annulations with suitable reaction partners to afford six-membered ring structures [37–47]. Recently, He and co-workers disclosed that the reaction between α-substituted allenoates and β,γ-unsaturated *N*-sulfonylimines proceeded in an unexpected [3 + 2] annulation mode to afford a cyclopentene ring with an all-carbon quaternary center (Scheme 1) [48]. In recent years our group has developed a family of amino acid-derived bifunctional phosphines and has intensively investigated related asymmetric transformations [49–63]. We became interested in developing an asymmetric variant of the above transformation by utilizing our amino acid-derived bifunctional phosphine catalysts.

## Results and Discussion

We chose the [3 + 2] annulation between α-benzyl-substituted allenoate **1a** and β,γ-unsaturated *N*-sulfonylimine **2a** as a model reaction and evaluated a number of amino acid based bifunctional phosphines as catalyst. As shown in Table 1, simple L-valine-derived phosphines **3a**–**c** were found to be effective in promoting the reaction, and products were obtained in moderate to good yields and with good *E*/*Z* ratios, and amide–phosphine **3b** worked best (Table 1, entries 2–4). L-Alanine-based phosphine **3d** and L-threonine-derived catalysts **3e** and **3f** did not provide better results (Table 1, entries 5–7). By employing L-threonine-derived catalyst **3g**, the enantioselectivity of the reaction was improved to 68%. To further improve the reaction results, we next utilized dipeptide phosphine catalysts, which are more structurally diverse and tunable. The L-thr-L-thr-derived catalyst **4a** was a poor catalyst, on the other hand, L-val-L-thr-derived catalyst **4b** led to adequately improved enantioselectivity of the reaction and was chosen for further investigations.

**Table 1:** Screening of different amino acid-based bifunctional phosphine catalysts.

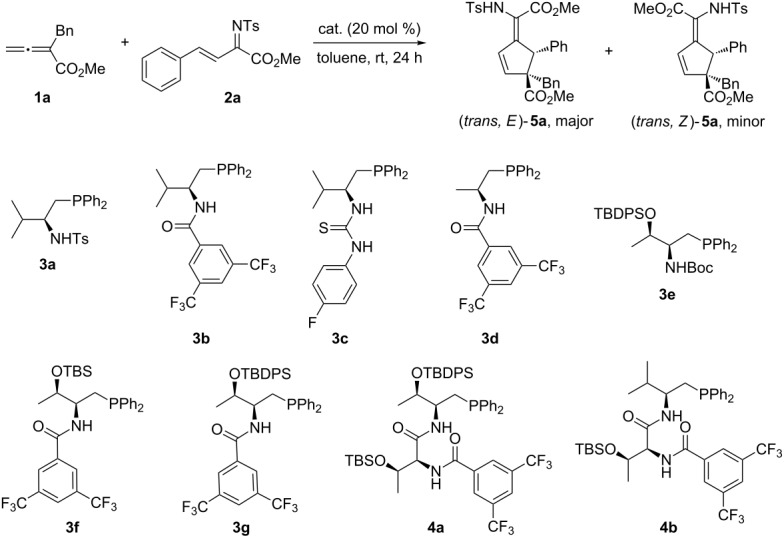				

Entry	Catalyst	*E*/*Z* ratio^a^	Yield (%)^b^	ee (%)^c^

1	MePPh_2_	85:15	67	–
2	**3a**	83:17	60	10
3	**3b**	88:12	70	48
4	**3c**	80:20	65	32
5	**3d**	89:11	72	35
6	**3e**	87:13	64	36
7	**3f**	85:15	73	47
8	**3g**	88:12	74	68
9	**4a**	86:14	71	60
10	**4b**	89:11	72	76

^a^Determined by ^1^H NMR analysis of the crude reaction mixture. ^b^Isolated yield of the *E-*isomers. ^c^Determined by HPLC analysis on a chiral stationary phase.

With the optimized conditions established, the substrate scope of this [3 + 2] annulation was explored by varying α-substituted allenoates **1** and imines **2** (Table 2). Firstly, different ester groups at the allenoates were examined (Table 2, entries 1–3). An allenoate bearing a *tert*-butyl ester group (**1b**) was found to be the best substrate, and the annulation products were obtained in good *E*/*Z* ratio, high yield and an ee of 84% (Table 2, entry 2). Allenoate substrates having different substitutions at the α-position were well tolerated, and the employment of various α-benzyl allenoates led to the formation of the products in consistently high *E*/*Z* ratios and enantioselectivities (Table 2, entries 4–6). It seemed that the presence of the *ortho* substituent in allenoates led to better enantioselectivity and decreased chemical yield (Table 2, entry 6). The utilization of 1-naphthyl substituted allenoate **1g** resulted in poor yield but excellent enantioselectivity (Table 2, entry 7). Notably, the electronic properties of the benzyl groups in allenoates did not have much effect on the reaction outcome (Table 2, entries 8 and 9). Furthermore, methoxycarbonylmethyl-substituted allenoate **1j** also proved to be a suitable substrate (Table 2, entry 10). The scope of β,γ-unsaturated *N*-sulfonylimines was subsequently examined by employing a number of differently substituted imines (Table 2, entries 11–15). In general, all the reactions worked well and afforded the annulation products in good *E*/*Z* ratios, moderate to good yields, and high enantioselectivities. Notably, imine **2e** bearing an electron rich aryl substituent was found to be a superior substrate; higher yield and ee value were attainable (Table 2, entry 14).

**Table 2:** Enantioselective [3 + 2] annulation of α-substituted allenoates with β,γ-unsaturated *N*-sulfonylimines catalyzed by dipeptide catalyst **4b**.^a^

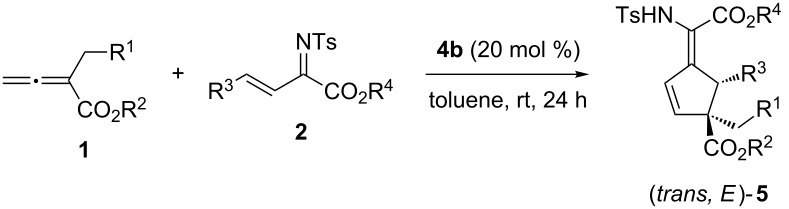						

Entry	**1** (R^1^/R^2^)	**2** (R^3^/R^4^)	**5**	*E/Z*^b^	Yield (%)^c^	ee (%)^d^

1	**1a** (Ph/Me)	**2a** (Ph/Me)	**5a**	89:11	76	76
2	**1b** (Ph/*t*-Bu)	**2a** (Ph/Me)	**5b**	83:17	70	84
3	**1c** (Ph/Bn)	**2a** (Ph/Me)	**5c**	85:15	72	78
4	**1d** (4-ClPh/*t*-Bu)	**2a** (Ph/Me)	**5d**	80:20	69	86
5	**1e** (3-ClPh/*t*-Bu)	**2a** (Ph/Me)	**5e**	81:19	60	89
6	**1f** (2-ClPh/*t*-Bu)	**2a** (Ph/Me)	**5f**	78:22	45	94
7	**1g** (1-naphthyl/*t*-Bu)	**2a** (Ph/Me)	**5g**	80:20	43	93
8	**1h** (4-MePh/*t*-Bu)	**2a** (Ph/Me)	**5h**	83:17	65	86
9	**1i** (4-NO_2_Ph/*t*-Bu)	**2a** (Ph/Me)	**5i**	81:19	73	92
10	**1j** (CO_2_Me/Bn)	**2a** (Ph/Me)	**5j**	90:10	72	82
11	**1b** (Ph/*t*-Bu)	**2b** (Ph/Et)	**5k**	80:20	68	85
12	**1b** (Ph/*t*-Bu)	**2c** (4-FPh/Et)	**5l**	83:17	55	86
13	**1b** (Ph/*t*-Bu)	**2d** (4-ClPh/Et)	**5m**	78:22	58	82
14	**1b** (Ph/*t*-Bu)	**2e** (4-MeOPh/Et)	**5n**	88:12	70	90
15	**1b** (Ph/*t*-Bu)	**2f** (2-Thienyl/Et)	**5o**	80:20	67	86

^a^Reactions were performed with **1** (0.15 mmol), **2** (0.1 mmol) and **4b** (0.02 mmol) in toluene (0.5 mL) at room temperature. ^b^Determined by ^1^H NMR analysis of the crude reaction mixture. ^c^Yield of isolated product. ^d^Determined by HPLC analysis on a chiral stationary phase.

**Scheme 2:**
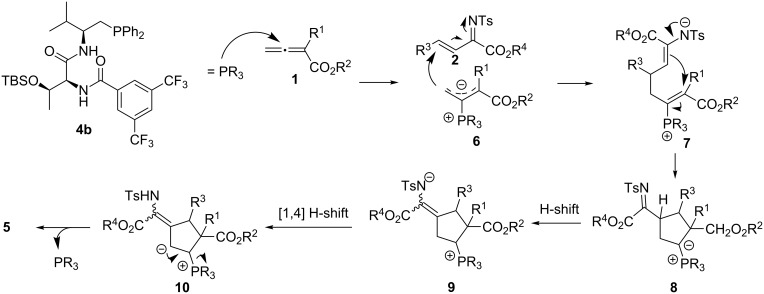
Possible reaction mechanism.

A possible reaction mechanism rationalizing the formation of the [3 + 2] annulation product is shown in Scheme 2 [34–36,48]. The reaction is initiated by the activation of the allenoate through a nucleophilic attack of the phosphine, generating zwitterionic intermediate **6**, which undergoes a [3 + 2] annulation with imine **2** to furnish intermediate **8**. Due to the lack of a hydrogen atom at the α-position, the normal proton shift in a typical [3 + 2] annulation cannot occur. Instead, this intermediate undergoes a proton shift to generate intermediate **9**, where a [1,4]-proton shift can occur to yield intermediate **10**. Lastly, elimination of the phosphine catalyst furnishes the final [3 + 2] annulation product **5**.

## Conclusion

In conclusion, we have described the first enantioselective [3 + 2] cycloaddition of α-substituted allenoates with β,γ-unsaturated *N*-sulfonylimines, catalyzed by amino acid-derived bifunctional phosphines. The [3 + 2] annulation reactions yielded highly functionalized cyclopentenes with an all-carbon quaternary center in moderate to good yields and good to excellent enantioselectivities. Further extension of the reaction reported herein and mechanistic studies are ongoing in our laboratory.

## Experimental

### General procedure for the [3 + 2] annulation

Into a flame-dried round bottle flask with a magnetic stirring bar under N_2_ at room temperature were added allenoate **1** (0.15 mmol) and β,γ-unsaturated *N*-sulfonylimine **2** (0.1 mmol), followed by the addition of anhydrous toluene (0.5 mL). Catalyst **4b** (0.02 mmol, 14.5 mg) was then introduced, and the reaction mixture was stirred at room temperature for 24 h. After complete consumption of the β,γ-unsaturated *N*-sulfonylimine, monitored by TLC, the solvent was removed under reduced pressure and the residue was purified by column chromatography on silica gel to afford annulation adducts **5**.

## Supporting Information

File 1 Additional material.
