# Supplementary material for: Enantioselective [3 + 2] annulation of α-substituted allenoates with β,γ-unsaturated N-sulfonylimines catalyzed by a bifunctional dipeptide phosphine
Source: Beilstein J Org Chem. 2016 Feb 24;12:343–8. doi: 10.3762/bjoc.12.37 (PMC4778533; doi:10.3762/bjoc.12.37)
Supplement: File 1 — Additional material. [file Beilstein_J_Org_Chem-12-343-s001.pdf]

Supporting Information

for

Enantioselective [3 + 2] annulation of α-substituted allenoates with β,γ-unsaturated *N*-sulfonylimines catalyzed by a bifunctional dipeptide phosphine

Huanzhen Ni, Weijun Yao and Yixin Lu*

Address: Department of Chemistry, National University of Singapore,

3 Science Drive 3, Singapore, 117543

Email: Yixin Lu - chmlyx@nus.edu.sg

*Corresponding author

Additional material

A. General information S2

B. Synthesis of dipeptide phosphine **4b** S2

C. Representative procedure S3

D. Analytical data of allenoates S4

E. Analytical data and HPLC chromatogram of the products S6

F. X-ray crystallographic analysis and determination of the absolute configurations of the products S28

G. References S30

H. NMR spectra of the products S31

A. General Information

Unless otherwise specified, all reactions were carried out under a nitrogen atmosphere in anhydrous conditions. All the solvents were purified according to the standard procedures. All chemicals which are commercially available were used without further purification unless otherwise noted. Thin-layer chromatography (TLC) was performed on silica gel plates (60F-254) using UV-light (254 and 365 nm). Flash chromatography was conducted on silica gel (300–400 mesh). ^{1}H and ^{13}C NMR spectra were recorded at ambient temperature in $CDCl_3$ on a Bruker AMX500 (500 MHz) spectrometer. Chemical shifts were reported in parts per million (ppm). All high resolution mass spectra were obtained on a Finnigan/MAT 95XL-T spectrometer. Optical rotations were measured using a Jasco DIP-1000 polarimeter. Enantiomeric excesses were determined by HPLC analysis on a chiral stationary phase.

Catalysts **3** & **4** were prepared by following our previously reported procedures [1]. The α-substituted alleoates were synthesized according to the reported procedures [2]. The β,γ-unsaturated *N*-sulfonylimines were prepared according the literature procedure from respective ketones [3].

B. Synthesis of dipeptide phosphine 4b

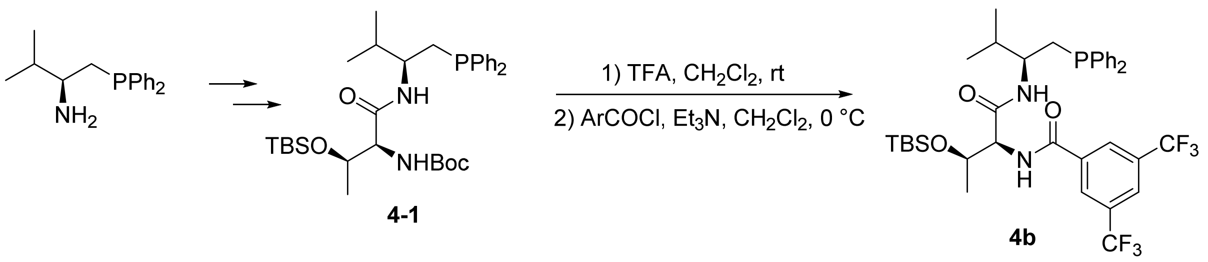

To a stirred solution of **4-1** [4] (1.17 g, 2 mmol) in anhydrous CH_2Cl_2 (20 mL) at room temperature was added TFA (4 mL). The resulting mixture was stirred for 2 h and then

quenched with saturated aqueous $NaHCO_3$ (100 mL). The product was extracted with CH_2Cl_2 several times (3 × 100 mL). The combined organic extracts were washed by brine (150 mL), dried over Na_2SO_4, filtered and concentrated. The residue was dissolved in anhydrous CH_2Cl_2 (20 mL) at 0 °C, Et_3N (5.6 mL, 4 mmol) was added, followed by 3,5-bis(trifluoromethyl)benzoyl chloride (550 mg, 2 mmol). After stirring at 0 °C for 1 h, solvent was removed and the residue was purified directly by flash column chromatography (hexane/ethyl acetate = 20 : 1) to afford **4b** as a white solid (871 mg, 60% yield).

^{1}H NMR (500 MHz, $CDCl_3$) δ 8.32 (s, 2H), 8.01 (s, 1H), 7.77 (d, *J* = 5.2 Hz, 1H), 7.52–7.41 (m, 4H), 7.41−7.29 (m, 6H), 7.10 (d, *J* = 9.1 Hz, 1H), 4.61−4.56 (m, 1H), 4.53−4.47 (m, 1H), 3.96 (qt, *J* = 9.1 Hz, 4.8 Hz, 1H), 2.35 (ddd, *J* = 13.9 Hz, 4.8 Hz, 1.7 Hz, 1H), 2.15 (ddd, *J* = 13.8 Hz, 9.3 Hz, 2.5 Hz, 1H), 2.04−1.96 (m, 1H), 1.31 (d, *J* = 6.3 Hz, 3H), 0.99 (s, 9H), 0.88 (dd, *J* = 6.7 Hz, 4.9 Hz, 6H), 0.29 (s, 3H), 0.21 (s, 3H); ^{13}C NMR (125 MHz, $CDCl_3$) δ 168.54, 164.25, 138.73, 138.63, 138.29, 138.18, 136.25, 133.26, 133.10, 132.77, 132.71, 132.56, 132.50, 132.23, 131.96, 129.09, 128.75, 128.73, 128.69, 128.67, 128.62, 127.65, 127.63, 126.31, 125.30, 125.27, 125.24, 124.14, 121.97, 119.80, 68.13, 58.23, 52.69, 52.57, 32.57, 32.50, 32.44, 25.98, 19.09, 18.07, 18.01, 17.96, 17.28, -4.71, -4.80; ^{31}P NMR (202 MHz, $CDCl_3$) δ -22.68. HRMS (ESI) m/z calcd for $C_{36}H_{46}F_6N_2O_3PSi$ $[M+H]^+$ = 727.2914, found = 727.2923.

C. Representative procedure

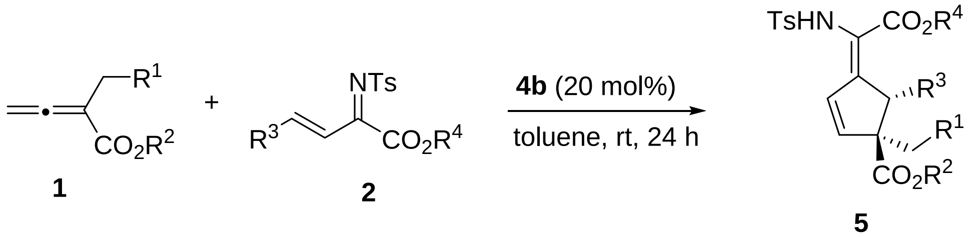

To a dried round bottle flask with a magnetic stirring bar under N_2 at room temperature were added allenoate **1** (0.15 mmol) and β,γ-unsaturated *N*-sulfonylimine **2** (0.1 mmol), followed by the addition of anhydrous toluene (0.5 mL). Catalyst **4b** (0.02 mmol, 14.5 mg) was then introduced, and the reaction mixture was stirred for 24 h. The solvent was removed under reduced pressure and the residue was purified by column chromatography on silica gel to afford annulation adducts **5**.

D. Analytical data of allenoates

tert-Butyl 2-(4-chlorobenzyl)buta-2,3-dienoate **1d**

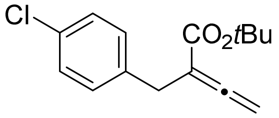

White solid; ^{1}H NMR (500 MHz, $CDCl_3$) δ 7.26 (d, *J* = 8.3 Hz, 2H), 7.17 (d, *J* = 8.3 Hz, 2H), 5.06 (d, *J* = 2.6 Hz, 2H), 3.50 (s, 2H), 1.45 (s, 9H); ^{13}C NMR (125 MHz, $CDCl_3$) δ 214.21, 165.84, 137.83, 132.02, 130.17, 128.32, 101.29, 81.23, 78.97, 34.33, 28.03; HRMS (EI) m/z calcd for $C_{15}H_{17}O_2Cl$ $[M]^+$ = 264.09171, found = 264.09105.

tert-Butyl 2-(3-chlorobenzyl)buta-2,3-dienoate **1e**

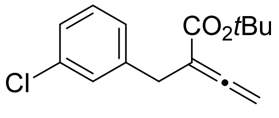

Colorless oil; ^{1}H NMR (500 MHz, $CDCl_3$) δ 7.27–7.17 (m, 3H), 7.14 (d, *J* = 7.0 Hz, 1H), 5.10 (t, *J* = 2.5 Hz, 2H), 3.52 (t, *J* = 2.5 Hz, 2H), 1.47 (s, 9H); ^{13}C NMR (125 MHz, $CDCl_3$) δ 214.27, 165.75, 141.40, 133.96, 129.44, 128.91, 127.02, 126.45, 101.04, 81.28, 79.02, 34.65, 28.02; HRMS (EI) m/z calcd for $C_{15}H_{17}O_2Cl$ $[M]^+$ = 264.09171, found = 264.09235.

tert-Butyl 2-(2-chlorobenzyl)buta-2,3-dienoate **1f**

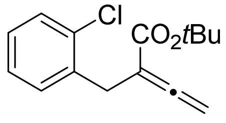

Colorless oil; ^{1}H NMR (500 MHz, $CDCl_3$) δ 7.36 (d, *J* = 7.5 Hz, 1H), 7.30 – 7.27 (m, 1H), 7.23–7.14 (m, 2H), 5.01 (t, *J* = 3.1 Hz, 2H), 3.68 (t, *J* = 3.0 Hz, 2H), 1.49 (s, 9H); ^{13}C NMR (126 MHz, $CDCl_3$) δ 214.17, 165.90, 136.73, 134.43, 130.84, 129.32, 127.73, 126.53, 100.29, 81.17, 79.17, 32.55, 28.05; HRMS (EI) m/z calcd for $C_{15}H_{17}O_2Cl$ $[M]^+$ = 264.09171 , found = 264.09212.

tert-Butyl 2-(naphthalen-1-ylmethyl)buta-2,3-dienoate **1g**

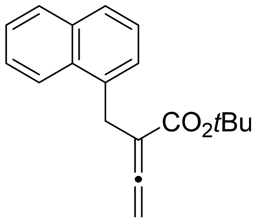

Colorless oil; ^{1}H NMR (300 MHz, $CDCl_3$) δ 8.09 – 8.00 (m, 1H), 7.85 (dd, *J* = 6.7 Hz, 2.7 Hz, 1H), 7.73 (dd, *J* = 6.2 Hz, 3.1 Hz, 1H), 7.56–7.37 (m, 4H), 4.83 (t, *J* = 3.1 Hz, 2H), 4.01 (t, *J* = 3.1 Hz, 2H), 1.49 (s, 9H); ^{13}C NMR (75 MHz, $CDCl_3$) δ 214.24, 166.11, 135.05, 133.73, 132.09, 128.51, 127.06, 126.93, 125.67, 125.32, 125.27, 124.21, 101.41, 81.07, 79.13, 31.84, 28.01; HRMS (EI) m/z calcd for $C_{19}H_{20}O_2$ $[M]^+$ = 280.14633, found = 280.14743.

tert-Butyl 2-(4-methylbenzyl)buta-2,3-dienoate **1h**

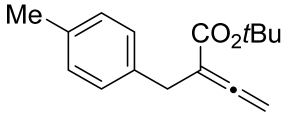

Colorless oil; ^{1}H NMR (500 MHz, $CDCl_3$) δ 7.15 (d, *J* = 7.9 Hz, 2H), 7.11 (d, *J* = 7.9 Hz, 2H), 5.06 (t, *J* = 2.5 Hz, 2H), 3.52 (s, 2H), 2.34 (s, 3H), 1.47 (s, 9H); ^{13}C NMR (125 MHz,

CDCl$_3$) δ 214.32, 166.13, 136.28, 135.66, 128.90, 128.70, 101.78, 81.00, 78.70, 34.49, 28.06, 21.06; HRMS (EI) m/z calcd for $C_{16}H_{20}O_2$ $[M]^+$ = 244.14633 , found = 244.14693.

tert-Butyl 2-(4-nitrobenzyl)buta-2,3-dienoate **1i**

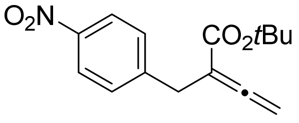

Colorless oil; ^{1}H NMR (500 MHz, CDCl$_3$) δ 8.13 (dd, *J* = 8.8 Hz, 2.2 Hz, 2H), 7.39 (d, *J* = 8.7 Hz, 2H), 5.10 (t, *J* = 2.1 Hz, 2H), 3.66–3.55 (m, 2H), 1.43 (s, 9H); ^{13}C NMR (126 MHz, CDCl$_3$) δ 214.22, 165.49, 147.21, 146.64, 129.61, 123.50, 100.37, 81.52, 79.34, 34.88, 27.98; HRMS (EI) m/z calcd for $C_{15}H_{17}O_4N$ $[M]^+$ = 275.11576 , found = 275.11545.

E. Analytical data and HPLC chromatogram of the products

Methyl (1*S*,5*S*,*E*)-1-benzyl-4-(2-methoxy-1-((4-methylphenyl)sulfonamido)-2-oxoethylidene)-5-phenylcyclopent-2-ene-1-carboxylate **5a**

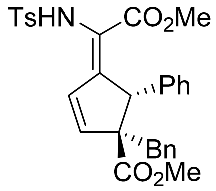

White solid; $[\alpha]^{25}{}_D$ = +237.1 (c 1.0, CHCl$_3$); ^{1}H NMR (500 MHz, CDCl$_3$) δ 7.54 (d, *J* = 8.1 Hz, 2H), 7.41−7.18 (m, 9H), 7.11 (d, *J* = 8.1 Hz, 2H), 6.98 (d, *J* = 7.5 Hz, 2H), 6.59 (d, *J* = 5.7 Hz, 1H), 6.14 (s, 1H), 5.06 (s, 1H), 3.62 (s, 3H), 3.07 (s, 3H), 2.71 (d, *J* = 13.5 Hz, 1H), 2.35 (s, 3H), 2.24 (d, *J* = 13.5 Hz, 1H); ^{13}C NMR (125 MHz, CDCl$_3$) δ 175.13, 164.32, 163.56, 146.94, 143.63, 140.19, 137.15, 135.95, 134.28, 129.62, 129.23, 128.23, 128.06, 127.56, 126.91, 126.71, 126.48, 115.45, 65.15, 56.24, 52.21, 51.34, 42.00, 21.45; HRMS (ESI) m/z calcd for $C_{30}H_{29}NNaO_6S$ $[M+Na]^+$ = 554.1608, found = 554.1616; The ee value

was 76%, t_R (major) = 24.993 min, t_R (minor) = 29.252 min (Chiralpak ID, λ = 254 nm, 40% *i*-PrOH/hexane, flow rate = 1.0 mL/min).

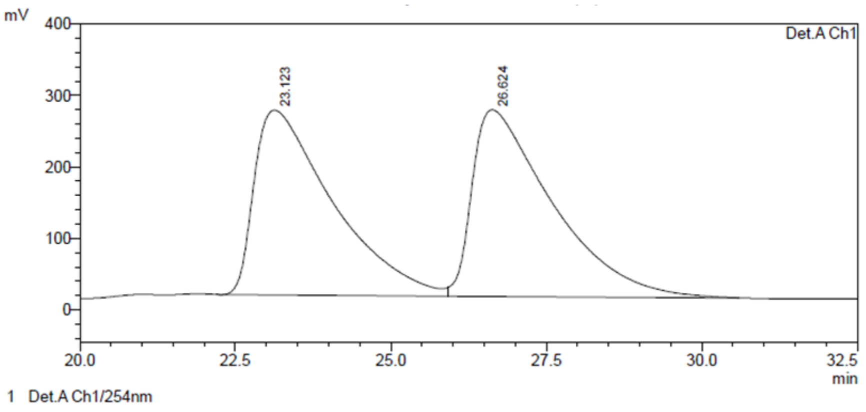

PeakTable

Detector A Ch1 254nm

Peak#	Ret. Time	Area	Height	Area %	Height %
1	23.123	22689966	258477	49.292	49.760
2	26.624	23341562	260971	50.708	50.240
Total		46031529	519448	100.000	100.000

Racemic **5a**

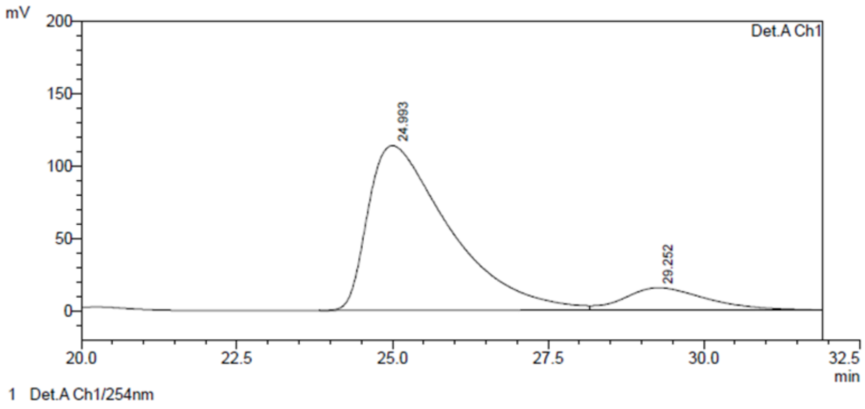

PeakTable

Detector A Ch1 254nm

Peak#	Ret. Time	Area	Height	Area %	Height %
1	24.993	10397675	113760	88.111	88.085
2	29.252	1403026	15388	11.889	11.915
Total		11800701	129148	100.000	100.000

Enantiomerically enriched **5a**

tert-Butyl (1S,5S,*E*)-1-benzyl-4-(2-methoxy-1-((4-methylphenyl)sulfonamido)-2-oxoethylidene)-5-phenylcyclopent-2-ene-1-carboxylate **5b**

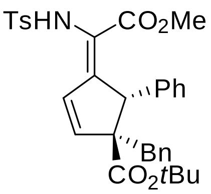

White solid; $[\alpha]^{25}_{D}$ = +170.7 (c 1.0, $CHCl_3$); ^{1}H NMR (500 MHz, $CDCl_3$) δ 7.53 (d, *J* = 8.2 Hz, 2H), 7.33 (t, *J* = 7.3 Hz, 3H), 7.28–7.16 (m, 6H), 7.11 (d, *J* = 8.2 Hz, 2H), 7.03 (d, *J* = 6.7 Hz, 2H), 6.58 (d, *J* = 5.7 Hz, 1H), 6.07 (s, 1H), 5.03 (s, 1H), 3.07 (s, 3H), 2.68 (d, *J* = 13.7 Hz, 1H), 2.34 (s, 3H), 2.30 (dd, *J* = 17.8 Hz, 4.0 Hz, 2H), 1.37 (s, 9H); ^{13}C NMR (125 MHz, $CDCl_3$) δ 173.60, 164.79, 163.65, 148.08, 143.57, 140.34, 137.42, 136.01, 133.82, 129.98, 129.20, 128.04, 127.94, 127.59, 126.76, 126.48, 115.18, 81.87, 65.51, 56.40, 51.29, 41.40, 27.90, 21.45; HRMS (ESI) m/z calcd for $C_{33}H_{35}NNaO_6S$ $[M+Na]^+$ = 596.2077, found = 596.2083; The ee value was 84%, t_R (minor) = 16.840 min, t_R (major) = 26.629 min (Chiralpak IC, λ = 254 nm, 20% *i*-PrOH/hexane, flow rate = 1.0 mL/min).

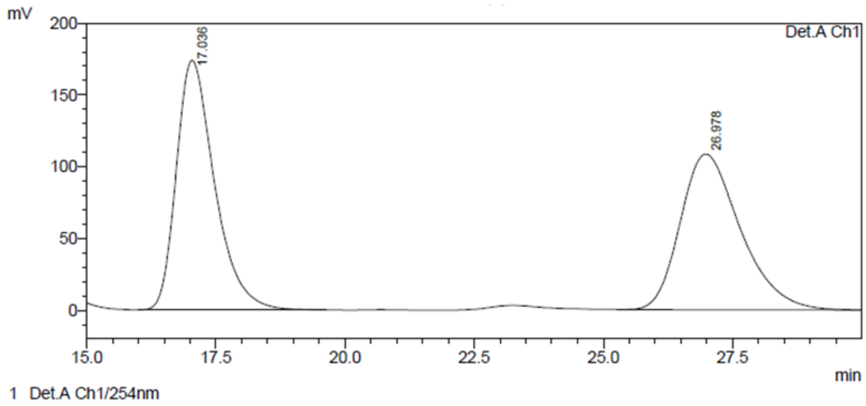

PeakTable

Detector A Ch1 254nm

Peak#	Ret. Time	Area	Height	Area %	Height %
1	17.036	8956143	173852	50.457	61.593
2	26.978	8793940	108409	49.543	38.407
Total		17750083	282261	100.000	100.000

Racemic **5b**

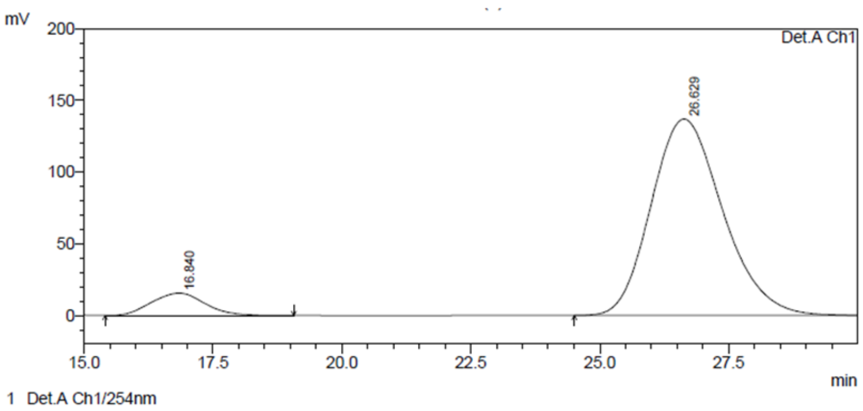

PeakTable

Detector A Ch1 254nm

Peak#	Ret. Time	Area	Height	Area %	Height %
1	16.840	1124220	15596	7.836	10.231
2	26.629	13222280	136847	92.164	89.769
Total		14346500	152444	100.000	100.000

Enantiomerically enriched **5b**

Benzyl (1*S*,5*S*,*E*)-1-benzyl-4-(2-methoxy-1-((4-methylphenyl)sulfonamido)-2-oxoethylidene)-5-phenylcyclopent-2-ene-1-carboxylate **5c**

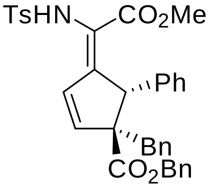

White solid; $[\alpha]^{25}{}_{D}$ = +135.3 (c 1.0, $CHCl_3$); ^{1}H NMR (500 MHz, $CDCl_3$) δ 7.52 (d, *J* = 8.1 Hz, 2H), 7.36 (t, *J* = 7.4 Hz, 2H), 7.32 (dd, *J* = 6.0 Hz, 3.6 Hz, 3H), 7.28 (t, *J* = 7.4 Hz, 2H), 7.20–7.13 (m, 5H), 7.09 (d, *J* = 8.2 Hz, 2H), 6.95 (dd, *J* = 6.2 Hz, 2.7 Hz, 2H), 6.61 (d, *J* = 5.8 Hz, 1H), 6.09 (s, 1H), 5.10–5.01 (m, 3H), 3.07 (s, 1H), 2.73 (d, *J* = 13.5 Hz, 1H), 2.34 (s, 1H), 2.28 (d, *J* = 13.5 Hz, 1H); ^{13}C NMR (125 MHz, $CDCl_3$) δ 174.48, 163.59, 146.87, 140.14, 137.07, 134.40, 129.71, 129.23, 128.50, 128.26, 128.22, 128.09, 127.56, 126.92, 126.66, 66.99, 65.17, 56.34, 51.34, 41.86, 21.45; HRMS (ESI) m/z calcd for $C_{36}H_{33}NNaO_6S$ $[M+Na]^+$ = 630.1921 , found = 630.1924; The ee value was 78%, t_R (minor) = 27.893 min, t_R (major) = 31.025 min (Chiralpak ID, λ = 254 nm, 40% *i*-PrOH/hexane, flow rate = 1.0 mL/min).

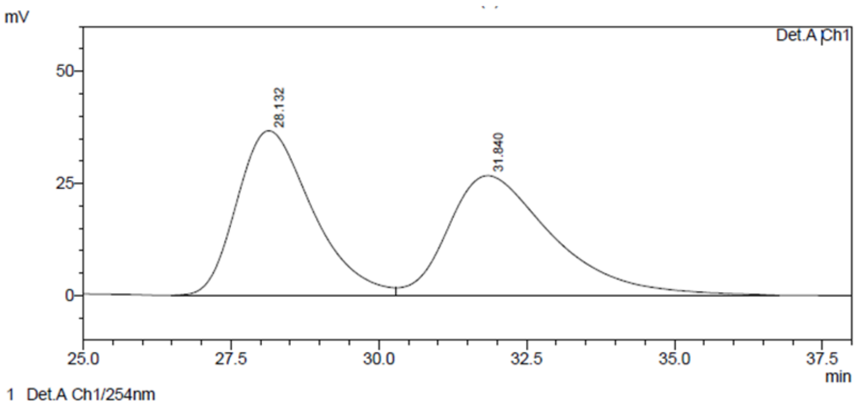

PeakTable

Detector A Ch1 254nm

Peak#	Ret. Time	Area	Height	Area %	Height %
1	28.132	3245618	36674	49.765	57.927
2	31.840	3276335	26637	50.235	42.073
Total		6521952	63312	100.000	100.000

Racemic **5c**

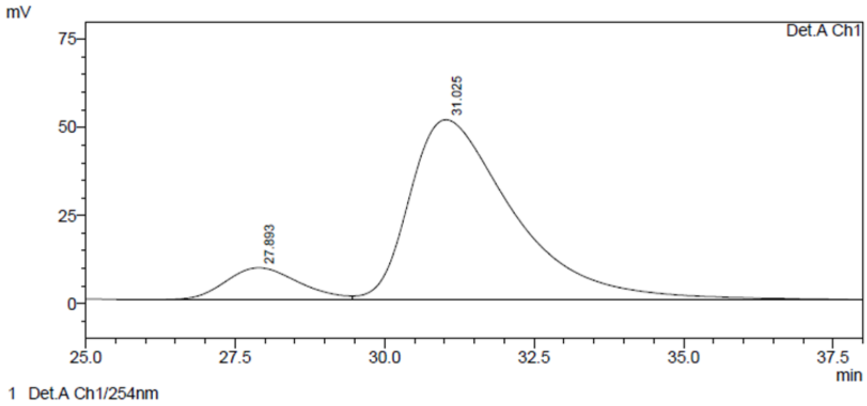

PeakTable

Detector A Ch1 254nm

Peak#	Ret. Time	Area	Height	Area %	Height %
1	27.893	768856	9030	11.056	15.028
2	31.025	6185377	51058	88.944	84.972
Total		6954233	60088	100.000	100.000

Enantiomerically enriched **5c**

tert-Butyl (1*S*,5*S*,*E*)-1-(4-chlorobenzyl)-4-(2-methoxy-1-((4-methylphenyl)sulfonamido)-2-oxoethylidene)-5-phenylcyclopent-2-ene-1-carboxylate **5d**

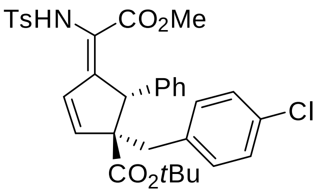

White solid; $[\alpha]^{25}{}_{D}$ = +165.2 (c 1.0, $CHCl_3$); ^{1}H NMR (500 MHz, $CDCl_3$) δ 7.52 (d, J = 8.2 Hz, 2H), 7.38–7.24 (m, 6H), 7.19 (d, J = 8.3 Hz, 2H), 7.10 (d, J = 8.2 Hz, 2H), 6.95 (d, J = 8.3 Hz, 2H), 6.50 (d, J = 5.7 Hz, 1H), 6.06 (s, 1H), 5.01 (s, 1H), 3.07 (s, 3H), 2.66 (d, J = 13.8 Hz, 1H), 2.34 (s, 3H), 2.30 (d, J = 13.8 Hz, 1H), 1.39 (s, 9H); ^{13}C NMR (125 MHz, $CDCl_3$) δ 173.37, 164.46, 163.56, 147.39, 143.60, 140.14, 135.98, 134.24, 132.40, 131.27, 129.21, 128.16, 127.57, 126.86, 115.36, 82.09, 77.27, 77.02, 76.77, 65.38, 56.28, 51.31, 40.53, 27.93, 21.45; HRMS (ESI) m/z calcd for $C_{33}H_{34}ClNNaO_6S$ $[M+Na]^+$ = 630.1688, found = 630.1692; The ee value was 86%, t_R (minor) = 15.961 min, t_R (major) = 25.349 min (Chiralpak IC, λ = 254 nm, 20% *i*-PrOH/hexane, flow rate = 1.0 mL/min).

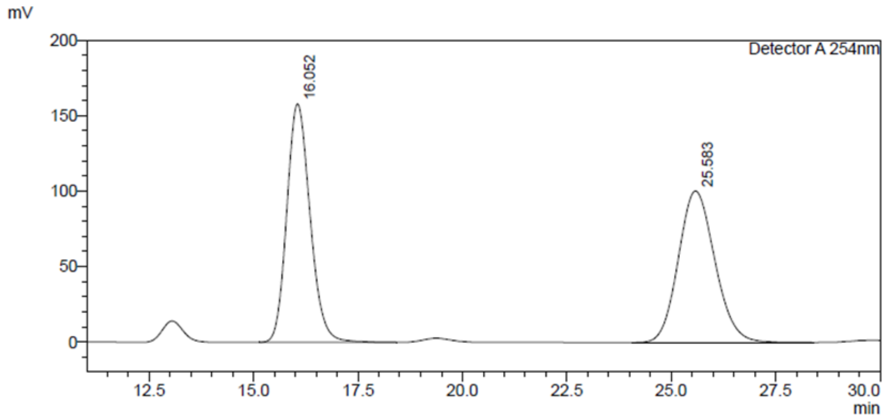

<Peak Table>

Detector A 254nm

Peak#	Ret. Time	Area	Height	Conc.	Unit	Mark	Name
1	16.052	6126171	157858	50.003			
2	25.583	6125323	100391	49.997		V	
Total		12251494	258249	100.000			

Racemic **5d**

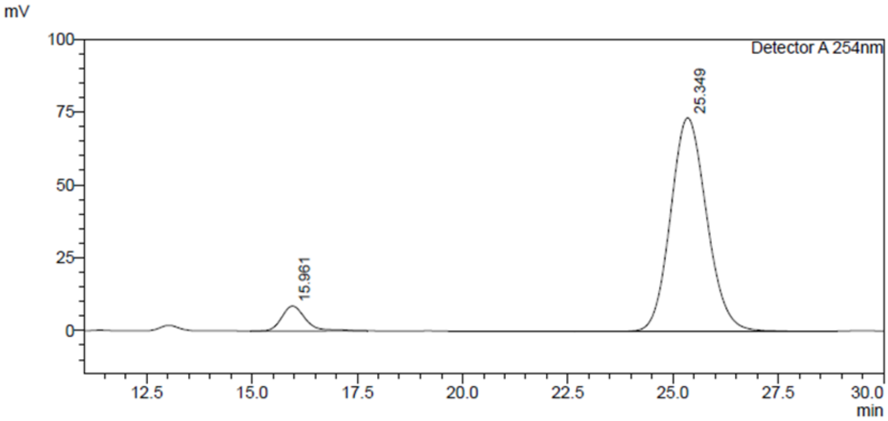

<Peak Table>

Detector A 254nm

Peak#	Ret. Time	Area	Height	Conc.	Unit	Mark	Name
1	15.961	331717	8505	7.050		M	
2	25.349	4373303	73247	92.950			
Total		4705020	81752	100.000			

Enantiomerically enriched **5d**

<u>*tert*-Butyl (1*S*,5*S*,*E*)-1-(3-chlorobenzyl)-4-(2-methoxy-1-((4-methylphenyl)sulfonamido)-2-oxoethylidene)-5-phenylcyclopent-2-ene-1-carboxylate</u> **5e**

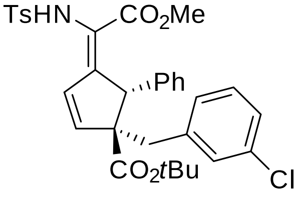

White solid; $[\alpha]^{25}{}_{D}$ = +155.5 (c 1.0, $CHCl_3$); ^{1}H NMR (500 MHz, $CDCl_3$) δ 7.52 (d, *J* = 8.2 Hz, 2H), 7.30 (dd, *J* = 25.2 Hz, 9.9 Hz, 6H), 7.16 (d, *J* = 7.2 Hz, 2H), 7.11 (d, *J* = 8.2 Hz, 2H), 7.01 (s, 1H), 6.90 (d, *J* = 6.7 Hz, 1H), 6.52 (d, *J* = 5.7 Hz, 1H), 6.08 (s, 1H), 5.03 (s, 1H), 3.07 (s, 3H), 2.66 (d, *J* = 13.8 Hz, 1H), 2.35 (s, 3H), 2.31 (d, *J* = 13.7 Hz, 1H), 1.41 (s, 9H); ^{13}C NMR (125 MHz, $CDCl_3$) δ 173.30, 164.38, 163.56, 147.32, 143.61, 140.10, 139.52, 135.99, 134.23, 133.86, 130.04, 129.27, 129.21, 128.08, 127.57, 126.90, 126.69, 115.41, 82.24, 65.35, 56.23, 51.32, 40.88, 27.92, 21.45; HRMS (ESI) m/z calcd for $C_{33}H_{34}ClNNaO_6S$ $[M+Na]^+$ = 630.1688, found = 630.1690; The ee value was 89%, t_R (minor) = 15.478 min, t_R (major) = 25.954 min (Chiralpak IC, λ = 254 nm, 20% *i*-PrOH/hexane, flow rate = 1.0 mL/min).

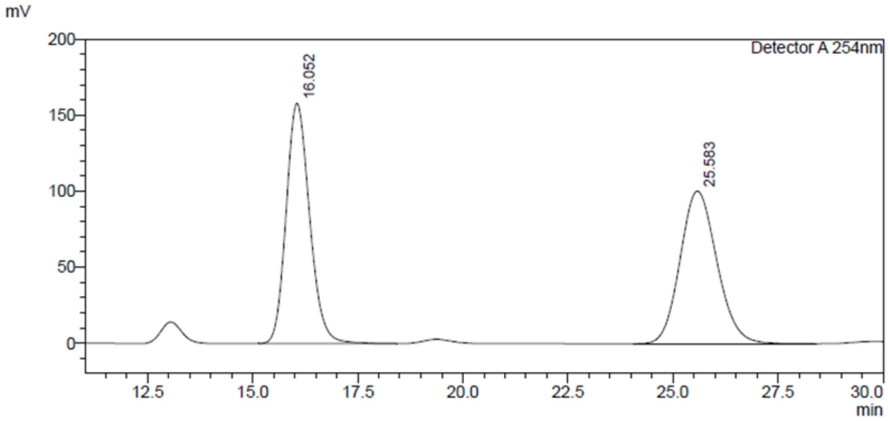

<Peak Table>

Detector A 254nm

Peak#	Ret. Time	Area	Height	Conc.	Unit	Mark	Name
1	16.052	6126171	157858	50.003			
2	25.583	6125323	100391	49.997		V	
Total		12251494	258249	100.000			

Racemic **5e**

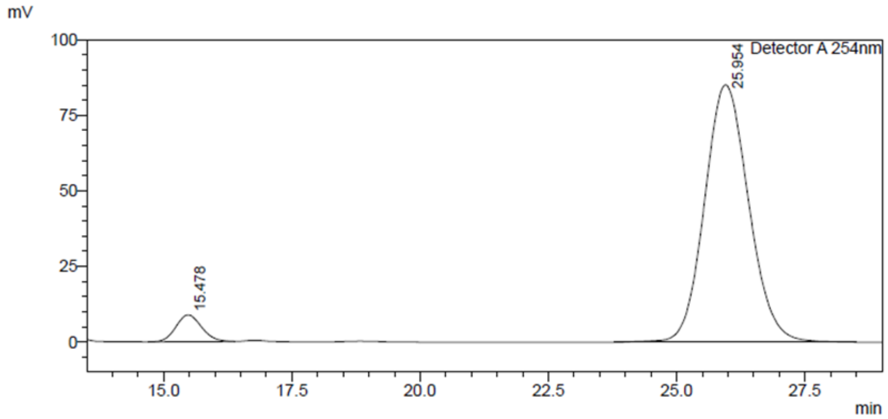

<Peak Table>

Detector A 254nm

Peak#	Ret. Time	Area	Height	Conc.	Unit	Mark	Name
1	15.478	305650	8828	5.656			
2	25.954	5098283	85106	94.344			
Total		5403933	93934	100.000			

Enantiomerically enriched **5e**

tert-Butyl (1*S*,5*S*,*E*)-1-(2-chlorobenzyl)-4-(2-methoxy-1-((4-methylphenyl)sulfonamido)-2-oxoethylidene)-5-phenylcyclopent-2-ene-1-carboxylate **5f**

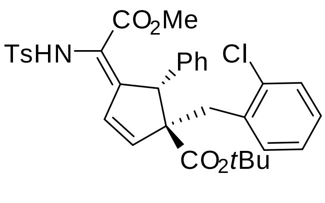

White solid; $[\alpha]^{25}{}_{D}$ = +123.3 (c 1.0, $CHCl_3$); ^{1}H NMR (500 MHz, $CDCl_3$) δ 7.50 (d, *J* = 8.1 Hz, 2H), 7.33 – 7.21 (m, 8H), 7.17–7.12 (m, 2H), 7.09 (d, *J* = 8.0 Hz, 2H), 6.51 (d, *J* = 5.6 Hz, 1H), 6.06 (s, 1H), 5.14 (s, 1H), 3.09 (s, 3H), 2.72 (q, *J* = 14.4 Hz, 2H), 2.34 (s, 3H), 1.36 (s, 9H); ^{13}C NMR (125 MHz, $CDCl_3$) δ 173.48, 164.55, 163.67, 148.23, 143.54, 140.20, 135.92, 135.85, 134.81, 133.76, 131.21, 129.53, 129.18, 127.86, 127.61, 126.88, 126.38, 115.07, 82.04, 65.59, 56.45, 51.31, 37.23, 27.83, 21.45; HRMS (ESI) m/z calcd for $C_{33}H_{34}ClNNaO_6S$ $[M+Na]^+$ = 630.1688, found = 630.1691; The ee value was 94%, t_R (minor) = 15.619 min, t_R (major) = 26.266 min (Chiralpak IC, λ = 254 nm, 20% *i*-PrOH/hexane, flow rate = 1.0 mL/min).

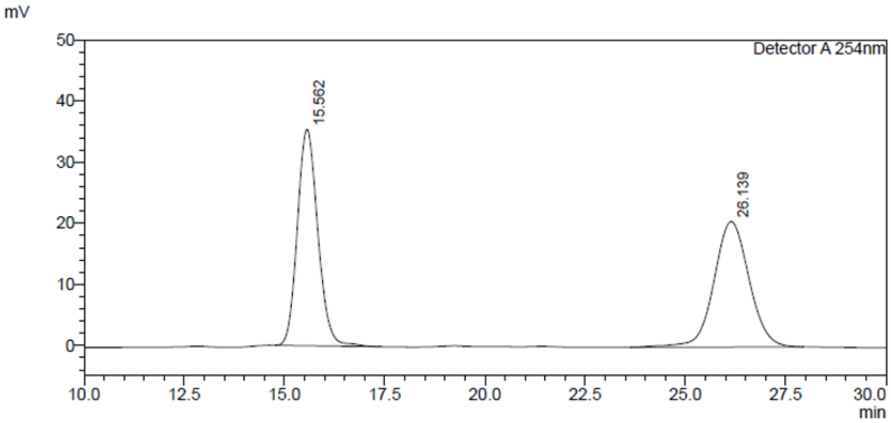

<Peak Table>

Detector A 254nm

Peak#	Ret. Time	Area	Height	Conc.	Unit	Mark	Name
1	15.562	1255985	35396	50.047			
2	26.139	1253642	20530	49.953			
Total		2509627	55926	100.000			

Racemic **5f**

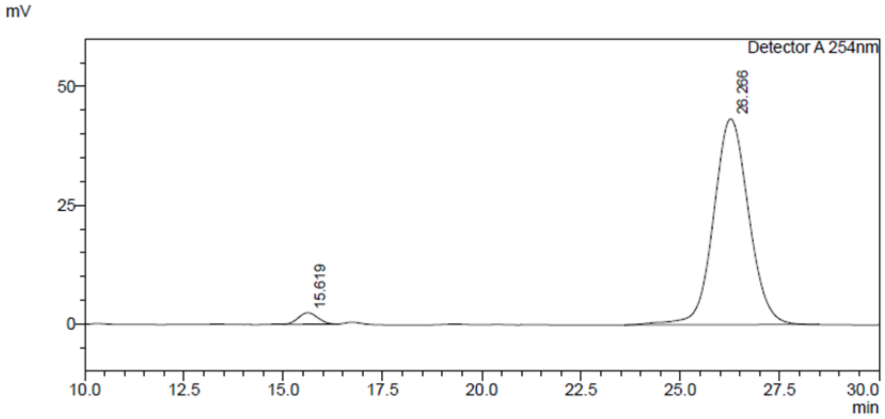

<Peak Table>

Detector A 254nm

Peak#	Ret. Time	Area	Height	Conc.	Unit	Mark	Name
1	15.619	82191	2420	2.983		V	
2	26.266	2673324	43331	97.017			
Total		2755515	45752	100.000			

Enantiomerically enriched **5f**

tert-Butyl (1*S*,5*S*,*E*)-4-(2-methoxy-1-((4-methylphenyl)sulfonamido)-2-oxoethylidene)-1-(naphthalen-1-ylmethyl)-5-phenylcyclopent-2-ene-1-carboxylate **5g**

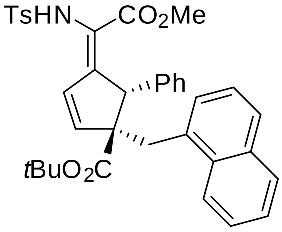

White solid; $[\alpha]^{25}_{D}$ = +180.1 (c 0.5, $CHCl_3$); ^{1}H NMR (500 MHz, $CDCl_3$) δ 7.82 (d, *J* = 7.6 Hz, 1H), 7.73 (t, *J* = 9.0 Hz, 2H), 7.55 (d, *J* = 8.2 Hz, 2H), 7.52–7.31 (m, 8H), 7.24 (dd, *J* = 17.5 Hz, 6.1 Hz, 2H), 7.12 (d, *J* = 8.1 Hz, 2H), 6.38 (d, *J* = 5.7 Hz, 1H), 6.07 (s, 1H), 5.18 (s, 1H), 3.10 (s, 3H), 3.05 (d, *J* = 14.4 Hz, 1H), 2.87 (d, *J* = 14.4 Hz, 1H), 2.35 (s, 3H), 1.24 (s, 9H); ^{13}C NMR (125 MHz, $CDCl_3$) δ 173.94, 164.62, 163.71, 148.83, 143.59, 140.74, 136.01, 133.92, 133.82, 133.44, 132.57, 129.22, 128.61, 127.81, 127.60, 127.38, 126.88, 125.94, 125.45, 125.05, 124.27, 115.15, 81.88, 66.11, 56.79, 51.33, 37.02, 27.76, 21.46; HRMS (ESI) m/z calcd for $C_{37}H_{37}NNaO_6S$ $[M+Na]^+$ = 646.2234, found = 646.2238; The ee value was

93%, t_R (minor) = 15.433 min, t_R (major) = 25.707 min (Chiralpak IC, λ = 254 nm, 20% *i*-PrOH/hexane, flow rate = 1.0 mL/min).

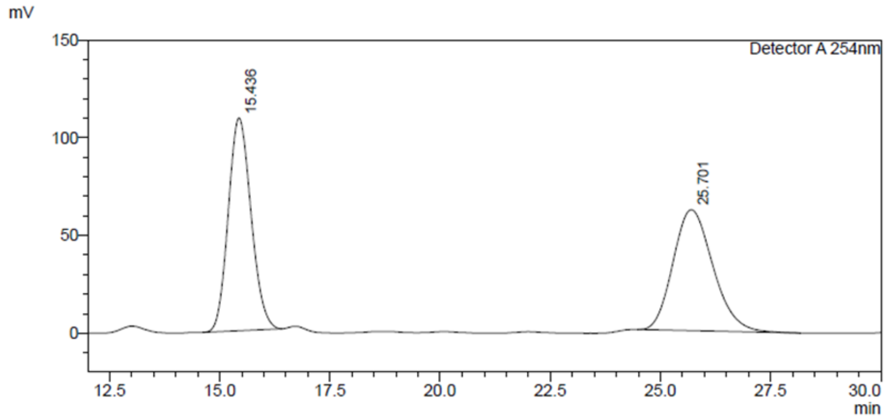

<Peak Table>

Detector A 254nm

Peak#	Ret. Time	Area	Height	Conc.	Unit	Mark	Name
1	15.436	3924722	108831	50.120			
2	25.701	3905950	61899	49.880			
Total		7830672	170730	100.000			

Racemic **5g**

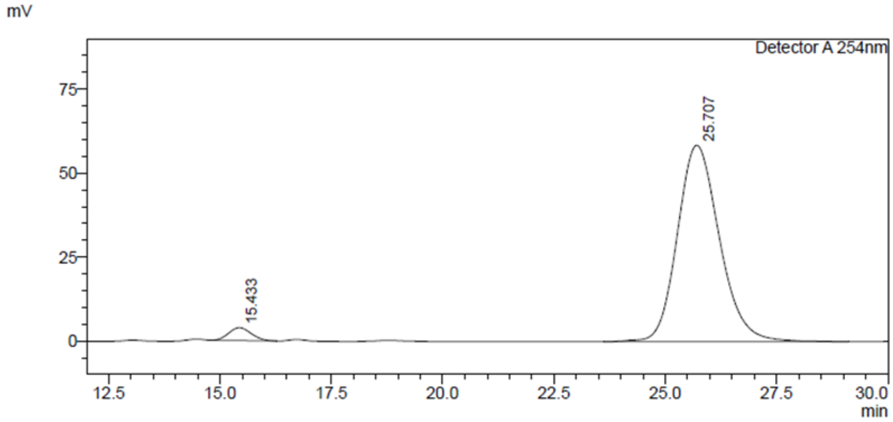

<Peak Table>

Detector A 254nm

Peak#	Ret. Time	Area	Height	Conc.	Unit	Mark	Name
1	15.433	133859	3810	3.358			
2	25.707	3851847	58502	96.642			
Total		3985706	62312	100.000			

Enantiomerically enriched **5g**

tert-Butyl (1*S*,5*S*,*E*)-4-(2-methoxy-1-((4-methylphenyl)sulfonamido)-2-oxoethylidene)-1-(4-methylbenzyl)-5-phenylcyclopent-2-ene-1-carboxylate **5h**

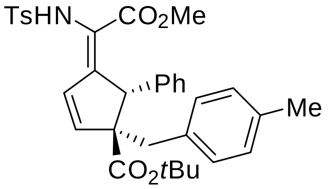

White solid; $[\alpha]^{25}{}_{D}$ = +152.3 (c 1.0, $CHCl_3$); ^{1}H NMR (500 MHz, $CDCl_3$) δ 7.53 (d, *J* = 8.2 Hz, 2H), 7.33 (t, *J* = 7.5 Hz, 3H), 7.25 (t, *J* = 7.2 Hz, 3H), 7.11 (d, *J* = 8.2 Hz, 2H), 7.02 (d, *J* = 7.9 Hz, 2H), 6.91 (d, *J* = 7.9 Hz, 2H), 6.56 (d, *J* = 5.7 Hz, 1H), 6.06 (s, 1H), 4.99 (s, 1H), 3.06 (s, 3H), 2.64 (d, *J* = 13.7 Hz, 1H), 2.34 (s, 3H), 2.30 (s, 3H), 2.24 (d, *J* = 13.8 Hz, 1H), 1.39 (s, 9H); ^{13}C NMR (125 MHz, $CDCl_3$) δ 173.69, 164.93, 163.66, 148.29, 143.55, 140.37, 136.03, 135.95, 134.22, 133.74, 129.85, 129.20, 128.71, 127.93, 127.59, 126.72, 115.10, 81.79, 65.57, 56.45, 51.27, 40.97, 27.94, 21.45, 20.99; HRMS (ESI) m/z calcd for $C_{34}H_{37}NNaO_6S$ $[M+Na]^+$ = 610.2234, found = 610.2237; The ee value was 86%, t_R (minor) = 16.601 min, t_R (major) = 24.594 min (Chiralpak IC, λ = 254 nm, 20% *i*-PrOH/hexane, flow rate = 1.0 mL/min).

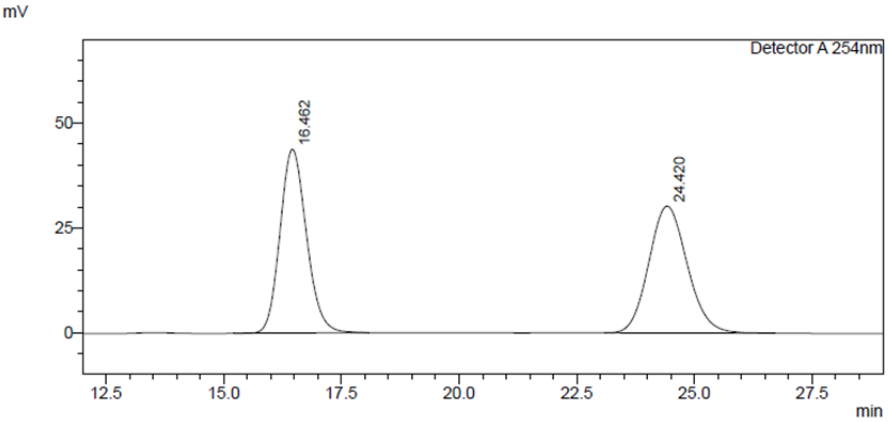

<Peak Table>

Detector A 254nm

Peak#	Ret. Time	Area	Height	Conc.	Unit	Mark	Name
1	16.462	1712770	43851	49.876			
2	24.420	1721261	30257	50.124			
Total		3434032	74108	100.000			

Racemic **5h**

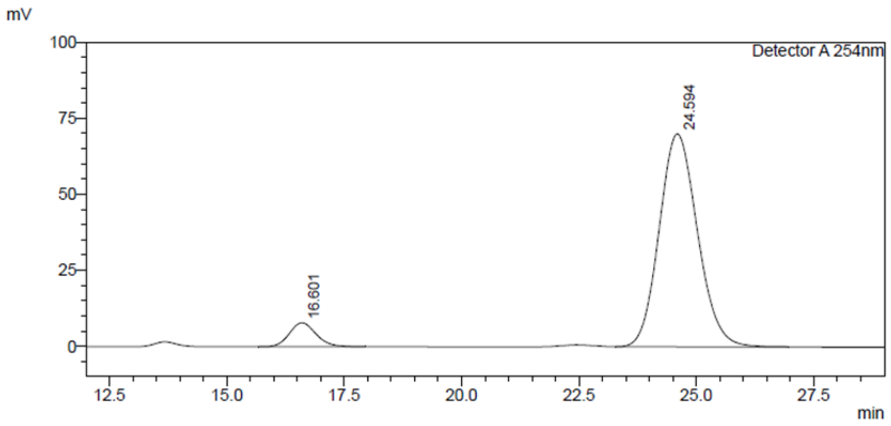

<Peak Table>

Detector A 254nm

Peak#	Ret. Time	Area	Height	Conc.	Unit	Mark	Name
1	16.601	309194	7843	7.235			
2	24.594	3964218	69929	92.765			
Total		4273412	77772	100.000			

Enantiomerically enriched **5h**

<u>*tert*-Butyl (1*S*,5*S*,*E*)-4-(2-methoxy-1-((4-methylphenyl)sulfonamido)-2-oxoethylidene)-1-(4-nitrobenzyl)-5-phenylcyclopent-2-ene-1-carboxylate **5i**</u>

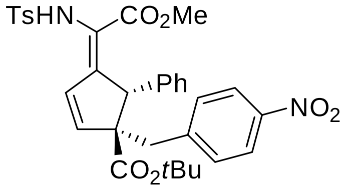

White solid; $[\alpha]^{25}{}_{D}$ = +150.4 (c 1.0, $CHCl_3$); ^{1}H NMR (500 MHz, $CDCl_3$) δ 8.07 (d, *J* = 8.6 Hz, 2H), 7.49 (d, *J* = 8.2 Hz, 2H), 7.32 (d, *J* = 5.7 Hz, 6H), 7.16 (d, *J* = 8.6 Hz, 2H), 7.10 (d, *J* = 8.1 Hz, 2H), 6.45 (d, *J* = 5.7 Hz, 1H), 6.10 (s, 1H), 5.07 (s, 1H), 3.07 (s, 3H), 2.81 (d, *J* = 13.9 Hz, 1H), 2.52 (d, *J* = 13.9 Hz, 1H), 2.34 (s, 3H), 1.39 (s, 9H); ^{13}C NMR (125 MHz, $CDCl_3$) δ 172.93, 163.93, 163.43, 146.75, 146.37, 145.59, 143.68, 139.84, 135.92, 134.90, 130.66, 129.23, 127.53, 127.08, 123.18, 115.72, 82.52, 65.23, 56.06, 51.37, 40.71, 27.92, 21.45; HRMS (ESI) m/z calcd for $C_{33}H_{34}N_2NaO_8S$ $[M+Na]^+$ = 641.1928, found = 641.1935; The ee value was 92%, t_R (minor) = 22.363 min, t_R (major) = 37.610 min (Chiralpak IC, λ = 254 nm, 40% *i*-PrOH/hexane, flow rate = 1.0 mL/min).

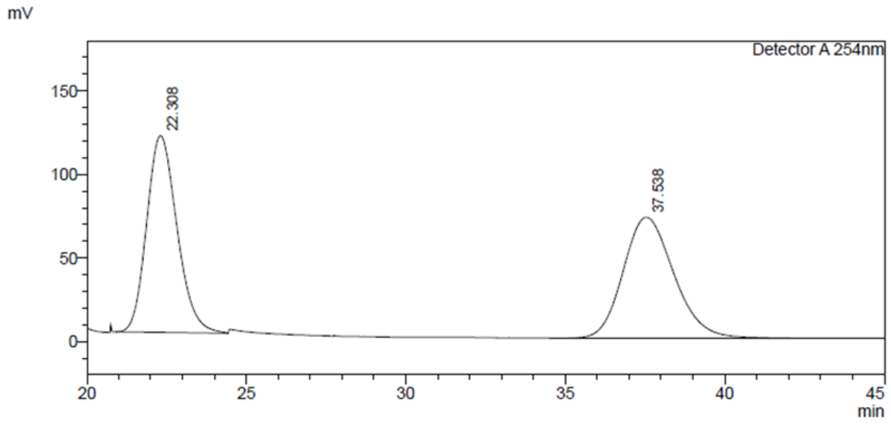

<Peak Table>

Detector A 254nm

Peak#	Ret. Time	Area	Height	Conc.	Unit	Mark	Name
1	22.308	7774552	117729	49.399			
2	37.538	7963851	72291	50.601			
Total		15738402	190020	100.000			

Racemic **5i**

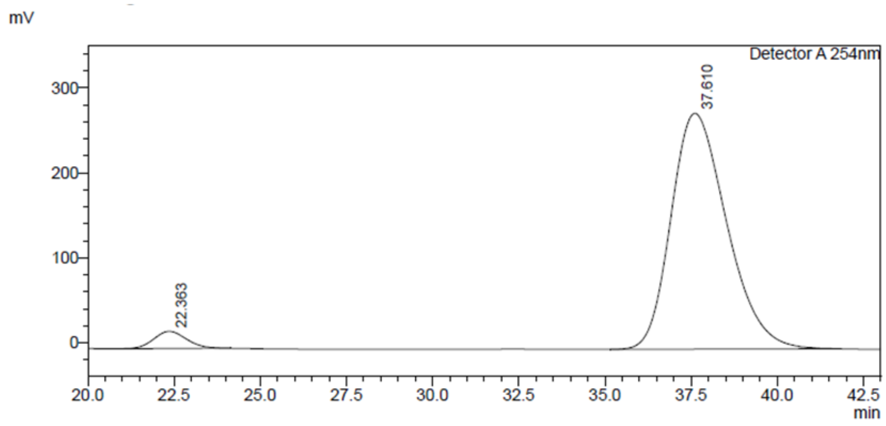

<Peak Table>

Detector A 254nm

Peak#	Ret. Time	Area	Height	Conc.	Unit	Mark	Name
1	22.363	1319126	20042	4.062		M	
2	37.610	31157584	277262	95.938		M	
Total		32476710	297303	100.000			

Enantiomerically enriched **5i**

Benzyl (1*S*,5*S*,*E*)-4-(2-methoxy-1-((4-methylphenyl)sulfonamido)-2-oxoethylidene)-1-(2-methoxy-2-oxoethyl)-5-phenylcyclopent-2-ene-1-carboxylate **5j**

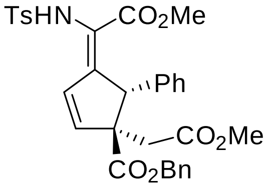

White solid; $[\alpha]^{25}{}_{D}$ = +38.1 (c 1, $CHCl_3$); ^{1}H NMR (500 MHz, $CDCl_3$) δ 7.44 (d, *J* = 8.2 Hz, 2H), 7.39 (d, *J* = 5.6 Hz, 1H), 7.31 (dt, *J* = 27.5 Hz, 5.7 Hz, 9H), 7.18 (s, 1H), 7.03 (d, *J* = 8.1 Hz, 2H), 6.53 (d, *J* = 5.6 Hz, 1H), 6.09 (s, 1H), 5.26 (s, 1H), 5.19 (s, 2H), 3.45 (s, 3H), 3.10 (s, 3H), 2.72 (d, *J* = 17.4 Hz, 1H), 2.34–2.26 (m, 4H); ^{13}C NMR (125 MHz, $CDCl_3$) δ 172.73, 171.28, 163.84, 163.53, 145.91, 143.59, 139.77, 136.62, 135.67, 129.18, 128.52, 128.23, 128.01, 127.60, 127.21, 116.10, 67.41, 62.39, 54.74, 51.53, 51.42, 37.61, 29.70, 21.43; HRMS (ESI) m/z calcd for $C_{32}H_{31}NNaO_8S$ $[M+Na]^+$ = 612.1663, found = 612.1667; The ee value was 82%, t_R (major) = 38.160 min, t_R (minor) = 47.679 min (Chiralpak IC, λ = 254 nm, 40% *i*-PrOH/hexane, flow rate = 1.0 mL/min).

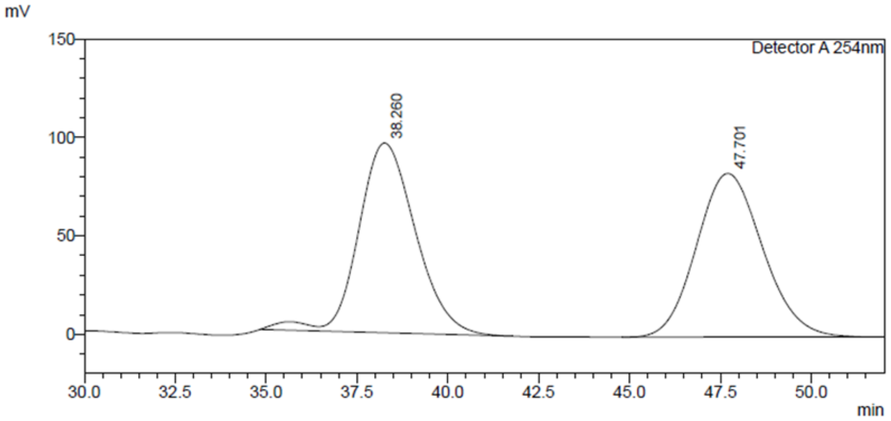

<Peak Table>

Detector A 254nm

Peak#	Ret. Time	Area	Height	Conc.	Unit	Mark	Name
1	38.260	10585895	96358	50.127		M	
2	47.701	10532107	83102	49.873		S	
Total		21118002	179459	100.000			

Racemic **5j**

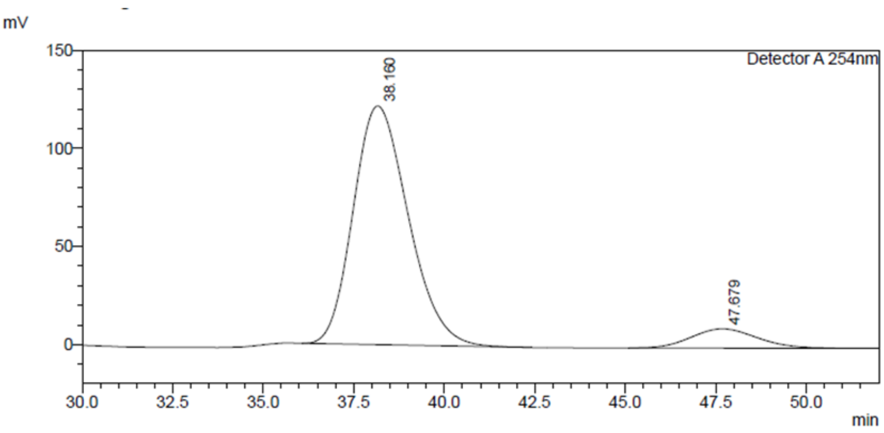

<Peak Table>

Detector A 254nm

Peak#	Ret. Time	Area	Height	Conc.	Unit	Mark	Name
1	38.160	12903005	121662	91.155			
2	47.679	1252059	9826	8.845			
Total		14155064	131488	100.000			

Enantiomerically enriched **5j**

<u>*tert*-Butyl (1*S*,5*S*,*E*)-1-benzyl-4-(2-ethoxy-1-((4-methylphenyl)sulfonamido)-2-oxoethylidene)-5-phenylcyclopent-2-ene-1-carboxylate **5k**</u>

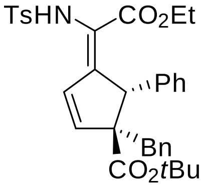

White solid; $[\alpha]^{25}{}_{D}$ = +201.9 (c 1.0, $CHCl_3$); ^{1}H NMR (500 MHz, CDCl3) δ 7.56 (d, *J* = 8.1 Hz, 2H), 7.38–7.16 (m, 9H), 7.11 (d, *J* = 8.2 Hz, 2H), 7.05 (d, *J* = 7.1 Hz, 2H), 6.56 (d, *J* = 5.7 Hz, 1H), 6.15 (s, 1H), 5.04 (s, 1H), 3.59 (ddd, *J* = 47.2 Hz, 10.8 Hz, 7.1 Hz, 2H), 2.70 (d, *J* = 13.7 Hz, 1H), 2.34 (s, 3H), 2.27 (d, *J* = 13.7 Hz, 1H), 1.39 (s, 9H), 0.75 (t, *J* = 7.1 Hz, 3H).

^{13}C NMR (125 MHz, $CDCl_3$) δ 173.65, 164.12, 163.33, 147.67, 143.54, 140.51, 137.46, 136.11, 134.00, 130.00, 129.23, 128.04, 127.63, 126.73, 126.48, 115.44, 81.80, 65.45, 60.79, 56.44, 41.47, 27.91, 21.41, 13.65; HRMS (ESI) m/z calcd for $C_{34}H_{37}NNaO_6S$ $[M+Na]^+$ =610.2234, found = 610.2240; The ee value was 85%, t_R (minor) = 15.748 min, t_R (major) = 19.359 min (Chiralpak IC, λ = 254 nm, 20% *i*-PrOH/hexane, flow rate = 1.0 mL/min).

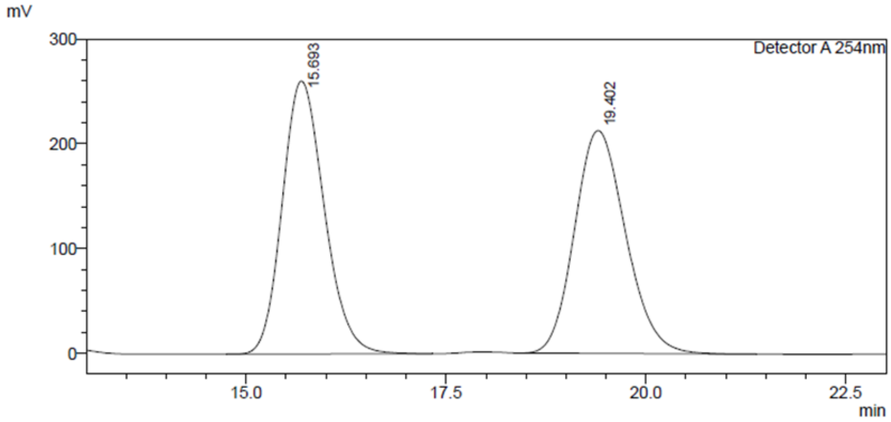

<Peak Table>

Detector A 254nm

Peak#	Ret. Time	Area	Height	Conc.	Unit	Mark	Name
1	15.693	9532599	260510	50.212			
2	19.402	9452135	212609	49.788			
Total		18984734	473118	100.000			

Racemic **5k**

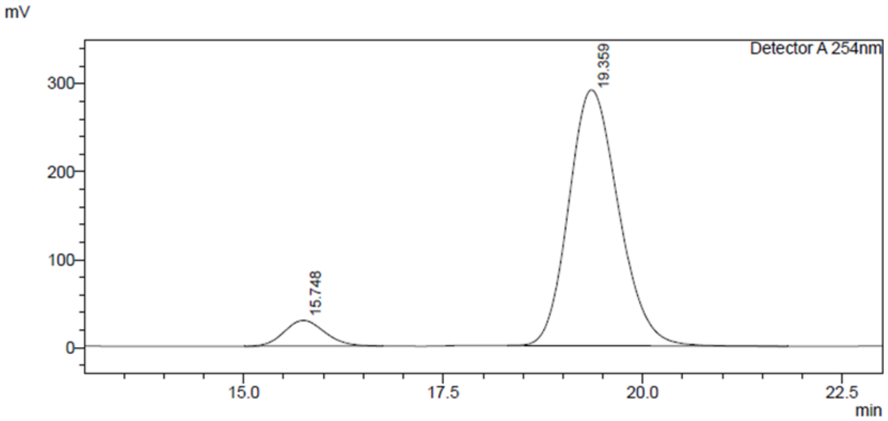

<Peak Table>

Detector A 254nm

Peak#	Ret. Time	Area	Height	Conc.	Unit	Mark	Name
1	15.748	1030519	29172	7.546		M	
2	19.359	12625397	290670	92.454			
Total		13655916	319842	100.000			

Enantiomerically enriched **5k**

tert-Butyl (1*S*,5*S*,*E*)-1-benzyl-4-(2-ethoxy-1-((4-methylphenyl)sulfonamido)-2-oxoethylidene)-5-(4-fluorophenyl)cyclopent-2-ene-1-carboxylate **5l**

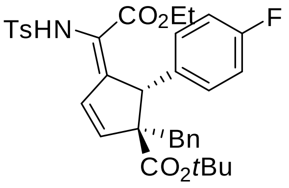

White solid; $[\alpha]^{25}_{D}$ = +200.0 (c 1.0, $CHCl_3$); ^1H NMR (500 MHz, $CDCl_3$) δ 7.53 (d, *J* = 8.2 Hz, 2H), 7.24 (ddd, *J* = 17.6 Hz, 15.5 Hz, 6.1 Hz, 6H), 7.14 (d, *J* = 8.2 Hz, 2H), 7.03 (d, *J* = 6.5 Hz, 4H), 6.56 (d, *J* = 5.7 Hz, 1H), 6.13 (s, 1H), 5.05 (s, 1H), 3.74–3.43 (m, 2H), 2.67 (d, *J* = 13.7 Hz, 1H), 2.36 (s, 2H), 2.30 (d, *J* = 13.7 Hz, 1H), 1.38 (s, 9H), 0.77 (t, *J* = 7.1 Hz, 3H); ^{13}C NMR (125 MHz, $CDCl_3$) δ 173.49, 164.19, 163.10, 147.71, 143.70, 137.23, 136.33, 136.16, 133.89, 129.88, 129.29, 128.09, 127.52, 126.56, 115.45, 114.79, 114.62, 81.95, 77.27, 77.02, 76.76, 65.43, 60.84, 55.47, 41.48, 27.89, 21.42, 13.68; HRMS (ESI) m/z calcd for $C_{34}H_{36}FNNaO_6S$ $[M+Na]^+$ =628.2140 , found = 628.2143; The ee value was 86%, t_R (minor) = 11.744 min, t_R (major) = 15.561 min (Chiralpak IC, λ = 254 nm, 20% *i*-PrOH/hexane, flow rate = 1.0 mL/min).

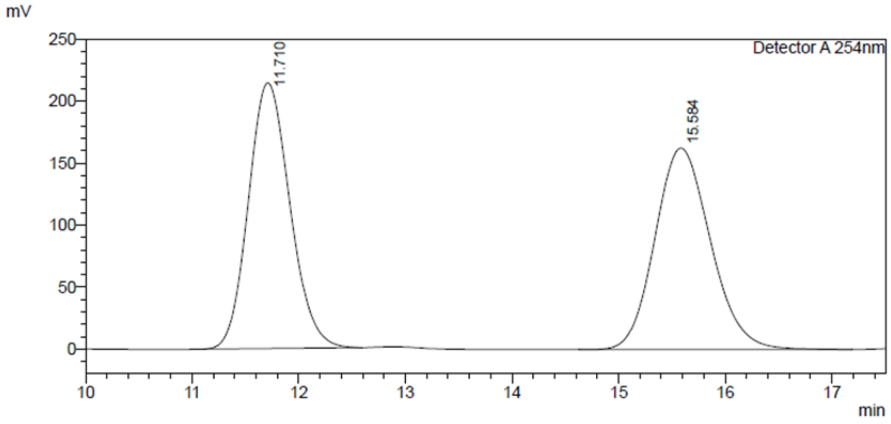

<Peak Table>

Detector A 254nm

Peak#	Ret. Time	Area	Height	Conc.	Unit	Mark	Name
1	11.710	5806861	214045	49.720			
2	15.584	5872293	162322	50.280			
Total		11679154	376367	100.000			

Racemic **5l**

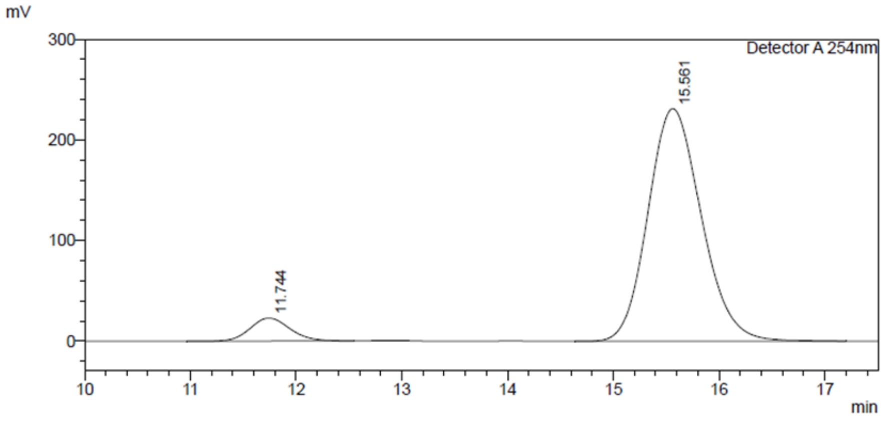

<Peak Table>

Detector A 254nm

Peak#	Ret. Time	Area	Height	Conc.	Unit	Mark	Name
1	11.744	598810	22885	6.876			
2	15.561	8109977	231270	93.124		M	
Total		8708787	254155	100.000			

Enantiomerically enriched **5l**

tert-Butyl (1*S*,5*S*,*E*)-1-benzyl-5-(4-chlorophenyl)-4-(2-ethoxy-1-((4-methylphenyl)sulfonamido)-2-oxoethylidene)cyclopent-2-ene-1-carboxylate **5m**

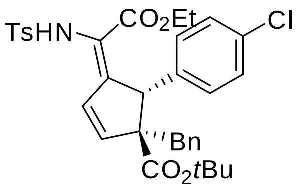

White solid; $[\alpha]^{25}{}_{D}$ = +176.3 (c 0.5, $CHCl_3$); ^{1}H NMR (500 MHz, $CDCl_3$) δ 7.52 (d, *J* = 8.2 Hz, 2H), 7.30 (s, 3H), 7.27 (d, *J* = 5.7 Hz, 1H), 7.21 (dd, *J* = 10.0 Hz, 7.1 Hz, 4H), 7.14 (d, *J* = 8.2 Hz, 2H), 7.06–6.98 (m, 2H), 6.55 (d, *J* = 5.7 Hz, 1H), 6.12 (s, 1H), 5.03 (s, 1H), 3.59 (ddq, *J* = 56.7 Hz, 10.8 Hz, 7.1 Hz, 2H), 2.67 (d, *J* = 13.7 Hz, 1H), 2.36 (s, 3H), 2.29 (d, *J* = 13.7 Hz, 1H), 1.37 (s, 9H), 0.78 (t, *J* = 7.1 Hz, 3H); ^{13}C NMR (125 MHz, $CDCl_3$) δ 173.40, 163.88, 163.04, 147.72, 143.75, 139.06, 137.13, 136.14, 133.89, 132.49, 129.88, 129.32, 128.10, 127.52, 126.59, 115.51, 82.01, 65.38, 60.88, 55.58, 41.51, 27.88, 21.44, 13.69; HRMS (ESI) m/z calcd for $C_{34}H_{36}ClNNaO_6S$ $[M+Na]^+$ = 644.1844, found = 644.1854; The ee value was 82%, t_R (minor) = 11.271 min, t_R (major) = 16.788 min (Chiralpak IC, λ = 254 nm, 20% *i*-PrOH/hexane, flow rate = 1.0 mL/min).

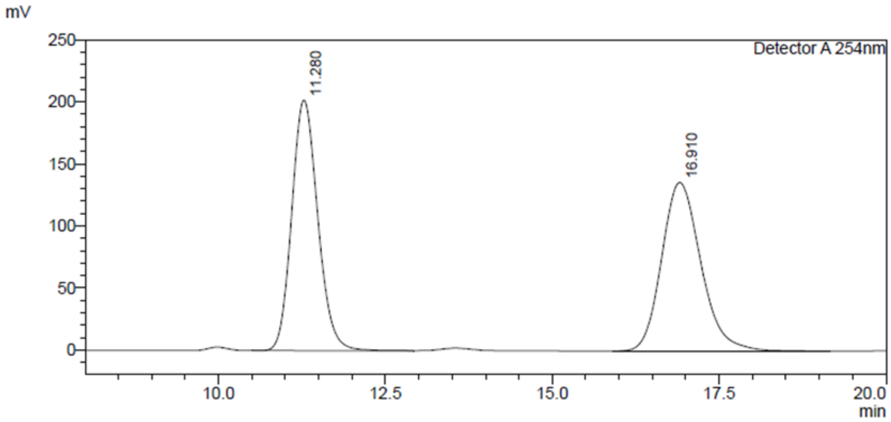

<Peak Table>

Detector A 254nm

Peak#	Ret. Time	Area	Height	Conc.	Unit	Mark	Name
1	11.280	5482797	201691	49.760			
2	16.910	5535700	135649	50.240			
Total		11018497	337340	100.000			

Racemic **5m**

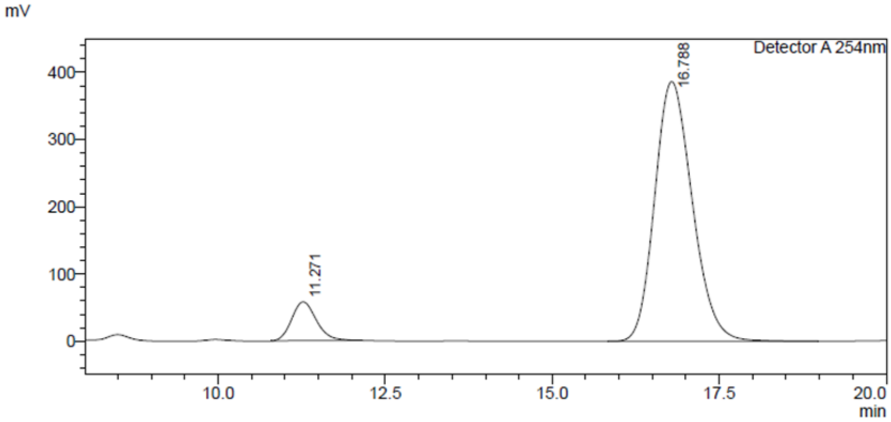

<Peak Table>

Detector A 254nm

Peak#	Ret. Time	Area	Height	Conc.	Unit	Mark	Name
1	11.271	1482866	57662	8.929		M	
2	16.788	15123872	386257	91.071			
Total		16606738	443919	100.000			

Enantiomerically enriched **5m**

tert-Butyl (1*S*,5*S*,*E*)-1-benzyl-4-(2-ethoxy-1-((4-methylphenyl)sulfonamido)-2-oxoethylidene)-5-(4-methoxyphenyl)cyclopent-2-ene-1-carboxylate **5n**

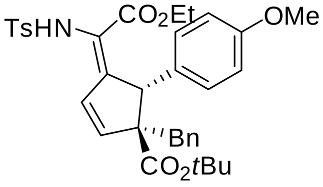

Yellow solid; $[\alpha]^{25}{}_{D}$ = +194.6 (c 1.0, $CHCl_3$); ^{1}H NMR (500 MHz, $CDCl_3$) δ 7.55 (d, *J* = 8.2 Hz, 2H), 7.27 (d, *J* = 5.8 Hz, 2H), 7.24 – 7.14 (m, 4H), 7.12 (d, *J* = 8.1 Hz, 2H), 7.04 (d, *J* = 6.9 Hz, 2H), 6.87 (d, *J* = 8.4 Hz, 2H), 6.53 (d, *J* = 5.7 Hz, 1H), 6.13 (s, 1H), 4.99 (s, 1H), 3.83 (s, 3H), 3.68–3.49 (m, 2H), 2.70 (d, *J* = 13.7 Hz, 1H), 2.34 (s, 3H), 2.26 (d, *J* = 13.8 Hz, 1H), 1.37 (s, 9H), 0.78 (t, *J* = 7.1 Hz, 3H); ^{13}C NMR (125 MHz, $CDCl_3$) δ 173.69, 164.52, 163.34, 158.37, 147.68, 143.54, 137.58, 136.19, 133.95, 132.77, 129.98, 129.24, 128.02, 127.62, 126.45, 115.27, 113.30, 81.71, 65.60, 60.79, 55.74, 55.29, 41.39, 27.90, 21.43, 13.72; HRMS (ESI) m/z calcd for $C_{35}H_{39}NNaO_7S$ $[M+Na]^+$ = 640.2339, found = 640.2337; The ee value was 90%, t_R (minor) = 19.172 min, t_R (major) = 34.647 min (Chiralpak IC, λ = 254 nm, 20% *i*-PrOH/hexane, flow rate = 1.0 mL/min).

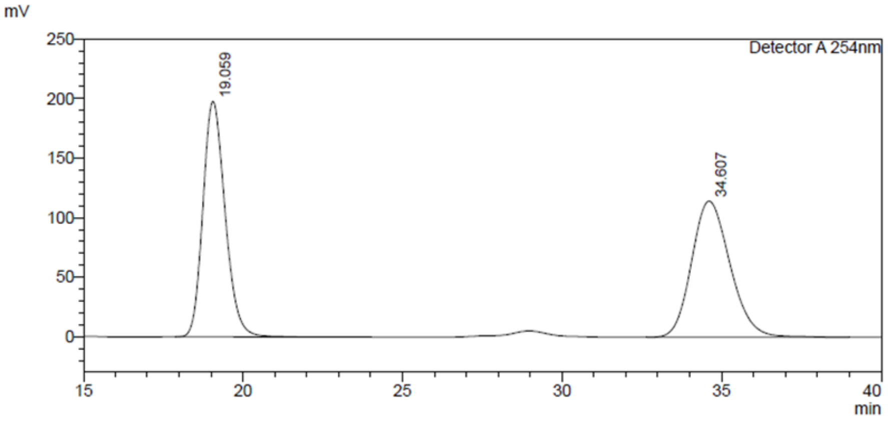

<Peak Table>

Detector A 254nm

Peak#	Ret. Time	Area	Height	Conc.	Unit	Mark	Name
1	19.059	9549288	197544	49.855			
2	34.607	9604949	114300	50.145		V	
Total		19154237	311844	100.000			

Racemic **5n**

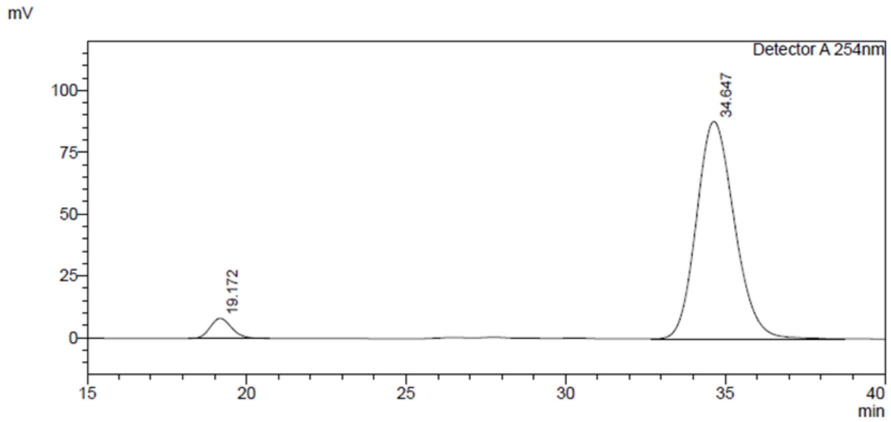

<Peak Table>

Detector A 254nm

Peak#	Ret. Time	Area	Height	Conc.	Unit	Mark	Name
1	19.172	381249	8126	4.975			
2	34.647	7282445	87874	95.025			
Total		7663694	96000	100.000			

Enantiomerically enriched **5n**

<u>*tert*-Butyl (1*S*,5*S*,*E*)-1-benzyl-4-(2-ethoxy-1-((4-methylphenyl)sulfonamido)-2-oxoethylidene)-5-(thiophen-2-yl)cyclopent-2-ene-1-carboxylate</u> **5o**

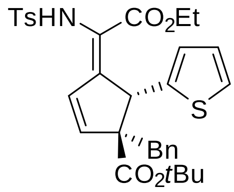

White solid; $[\alpha]^{25}{}_{D}$ = +87.9 (c 1.0, $CHCl_3$); ^{1}H NMR (500 MHz, $CDCl_3$) δ 7.55 (d, *J* = 8.2 Hz, 2H), 7.26 – 7.17 (m, 5H), 7.15 (d, *J* = 8.2 Hz, 2H), 7.08 (d, *J* = 6.8 Hz, 2H), 6.96 (d, *J* = 3.3 Hz, 2H), 6.51 (d, *J* = 5.8 Hz, 1H), 6.11 (s, 1H), 5.40 (s, 1H), 3.80–3.48 (m, 2H), 2.95 (d, *J* = 13.9 Hz, 1H), 2.59 (d, *J* = 13.9 Hz, 1H), 2.36 (s, 3H), 1.36 (s, 9H), 0.86 (t, *J* = 7.1 Hz, 3H); ^{13}C NMR (125 MHz, $CDCl_3$) δ 172.91, 163.43, 162.69, 146.96, 143.59, 142.43, 137.43, 136.10, 133.39, 129.89, 129.28, 128.06, 127.68, 126.49, 126.16, 123.71, 115.67, 81.94, 65.75, 61.03, 51.42, 39.92, 27.85, 21.45, 13.76; HRMS (ESI) m/z calcd for $C_{32}H_{35}NNaO_6S_2$ $[M+Na]^+$ = 616.1798, found = 616.1802; The ee value was 86%, t_R (minor) = 17.527 min, t_R (major) = 26.310 min (Chiralpak IC, λ = 254 nm, 20% *i*-PrOH/hexane, flow rate = 1.0 mL/min).

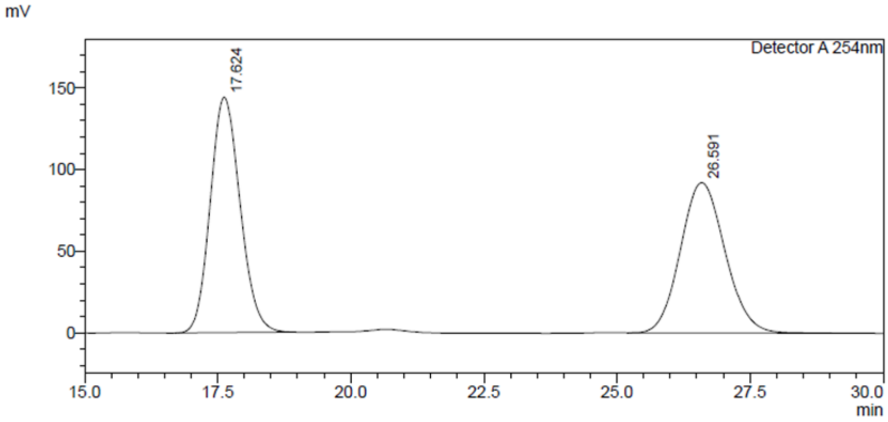

<Peak Table>

Detector A 254nm

Peak#	Ret. Time	Area	Height	Conc.	Unit	Mark	Name
1	17.624	5639967	144219	51.198		M	
2	26.591	5376016	92072	48.802			
Total		11015983	236292	100.000			

Racemic **5o**

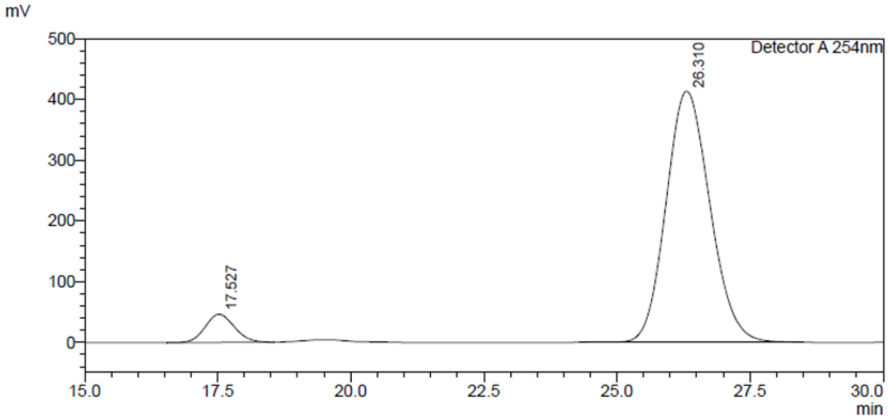

<Peak Table>

Detector A 254nm

Peak#	Ret. Time	Area	Height	Conc.	Unit	Mark	Name
1	17.527	1760653	46172	6.908			
2	26.310	23727009	413407	93.092		M	
Total		25487662	459579	100.000			

Enantiomerically enriched **5o**

F. X-Ray crystallographic analysis and determination of the absolute configurations of the products

X-Ray crystallographic analysis of 5a

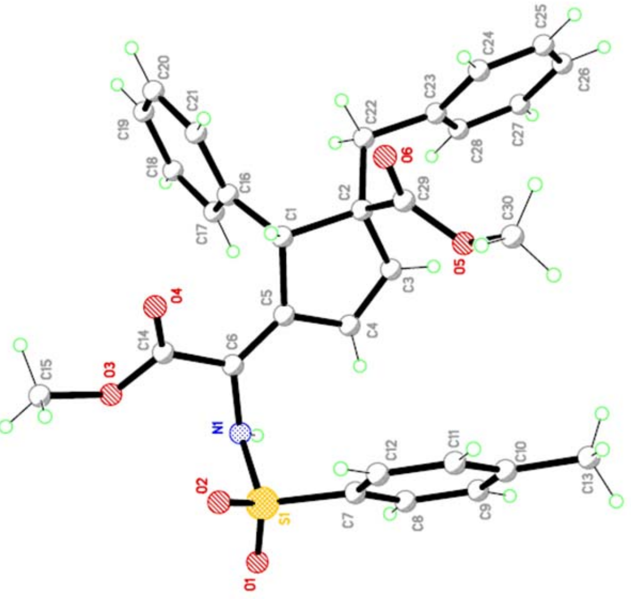

Figure 1. X-ray structure of **5a**

Table 1. Crystal data and structure refinement for f562.

Identification code	f562	
Empirical formula	C30 H29 N O6 S	
Formula weight	531.60	
Temperature	100(2) K	
Wavelength	1.54178 Å	
Crystal system	Monoclinic	
Space group	$P2_1$	
Unit cell dimensions	a = 6.2607(4) Å	α= 90°.
	b = 20.9073(12) Å	β= 100.7453(16)°.
	c = 10.4305(6) Å	γ = 90°.
Volume	1341.35(14) $Å^3$	
Z	2	
Density (calculated)	1.316 Mg/m^3	
Absorption coefficient	1.444 mm^{-1}	
F(000)	560	
Crystal size	0.514 x 0.120 x 0.094 mm^3	
Theta range for data collection	4.229 to 78.516°.	
Index ranges	-7<=h<=7, -26<=k<=25, -12<=l<=13	
Reflections collected	16251	
Independent reflections	5317 [R(int) = 0.0504]	
Completeness to theta = 67.679°	99.6 %	

Absorption correction	Semi-empirical from equivalents
Max. and min. transmission	0.7531 and 0.4820
Refinement method	Full-matrix least-squares on F^2
Data / restraints / parameters	5317 / 1 / 350
Goodness-of-fit on F^2	1.083
Final R indices [I>2sigma(I)]	R1 = 0.0535, wR2 = 0.1373
R indices (all data)	R1 = 0.0545, wR2 = 0.1398
Absolute structure parameter	0.106(9)
Extinction coefficient	n/a
Largest diff. peak and hole	0.477 and -0.632 e.$Å^{-3}$

G. References

[1] a) Han, X.; Wang, Y.; Zhong, F.; Lu, Y. *J. Am. Chem. Soc.* **2011**, *133*, 1726; b) Han, X.; Zhong, F.; Wang, Y.; Lu, Y. *Angew. Chem. Int. Ed.* **2012**, *51*, 767; c) Zhong, F.; Han, X.; Wang, Y.; Lu, Y. *Chem. Sci.* **2012**, *3*, 1231; d) Zhong, F.; Han, X.; Wang, Y.; Lu, Y. *Angew. Chem. Int. Ed.* **2011**, *50*, 7837; e) Zhong, F.; Luo, J.; Chen, G.-Y.; Dou, X.; Lu, Y. *J. Am. Chem. Soc.* **2012**, *134*, 10222; f) Zhong, F.; Dou, X.; Han, X.; Yao, W.; Zhu, Q.; Meng, Y.; Lu, Y. *Angew. Chem. Int. Ed.* **2013**, *52*, 943; g) Yao, W.; Dou, X.; Lu, Y. *J. Am. Chem. Soc.* **2015**, *137*, 54.

[2] Zhu, X.-F.; Lan, J.; Kwon, O. *J. Am. Chem. Soc.* **2003**, *125*, 4716;

[3] Lu, L.-Q.; Zhang, J.-J.; Li, F.; Cheng, Y.; An, J.; Chen, J.-R.; Xiao, W.-J. *Angew. Chem., Int. Ed.* **2010**, *49*, 4495

[4] Wang, T.; Yao, W.; Zhong, F.; Pang, G. H.; Lu, Y. *Angew. Chem. Int. Ed.* **2014**, *53*, 2964.

H. NMR spectra of the products

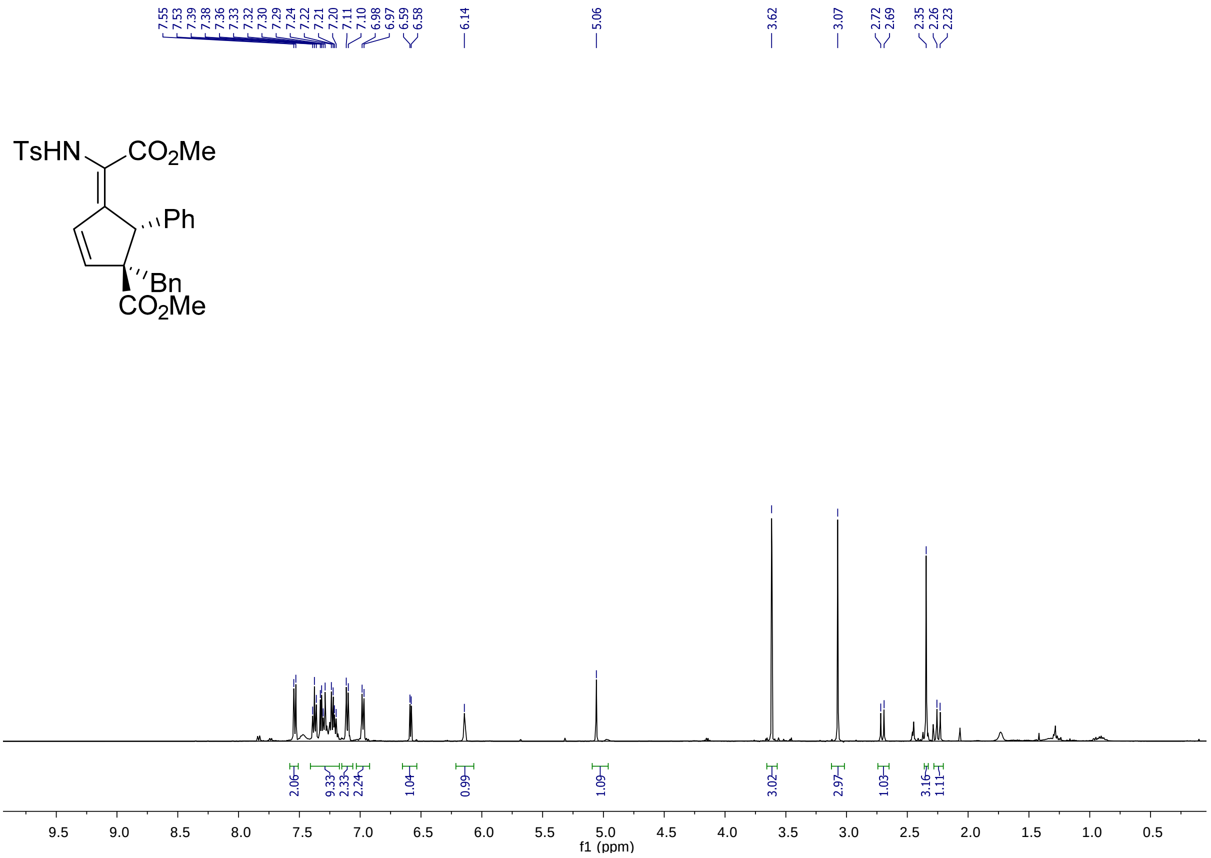

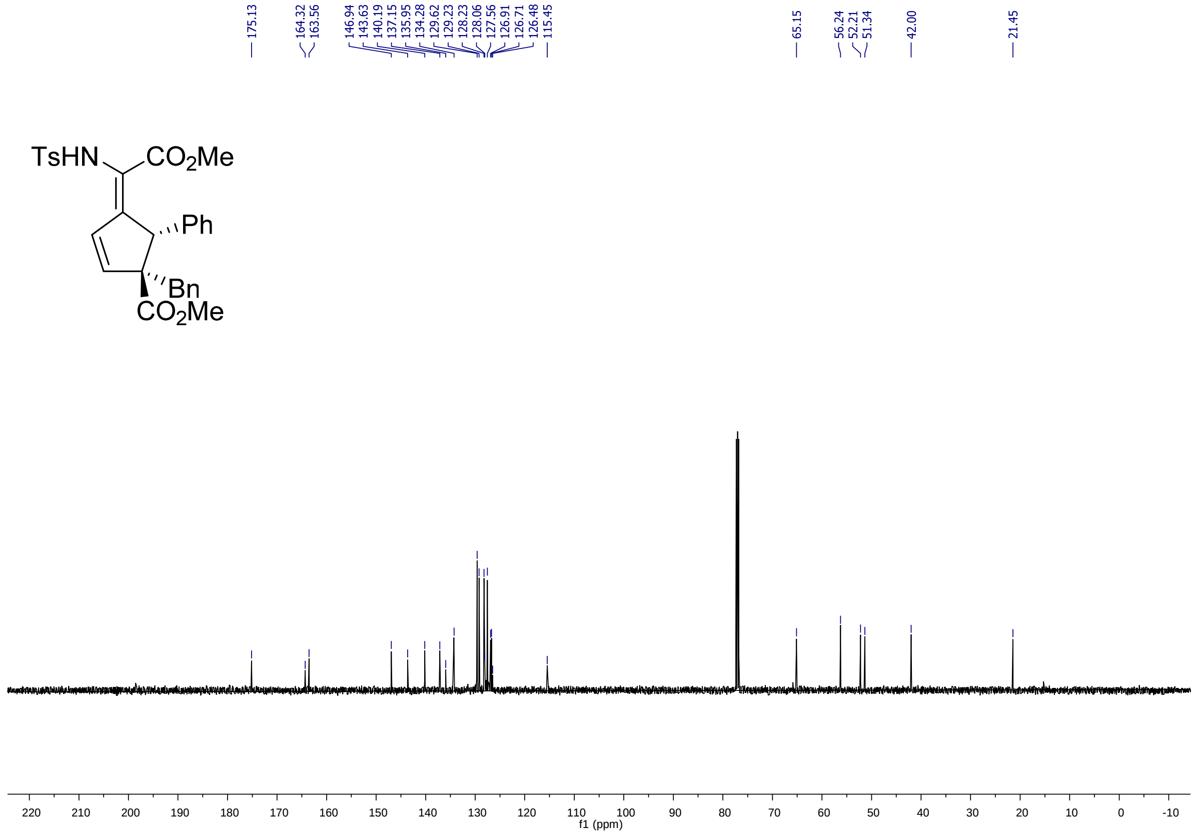

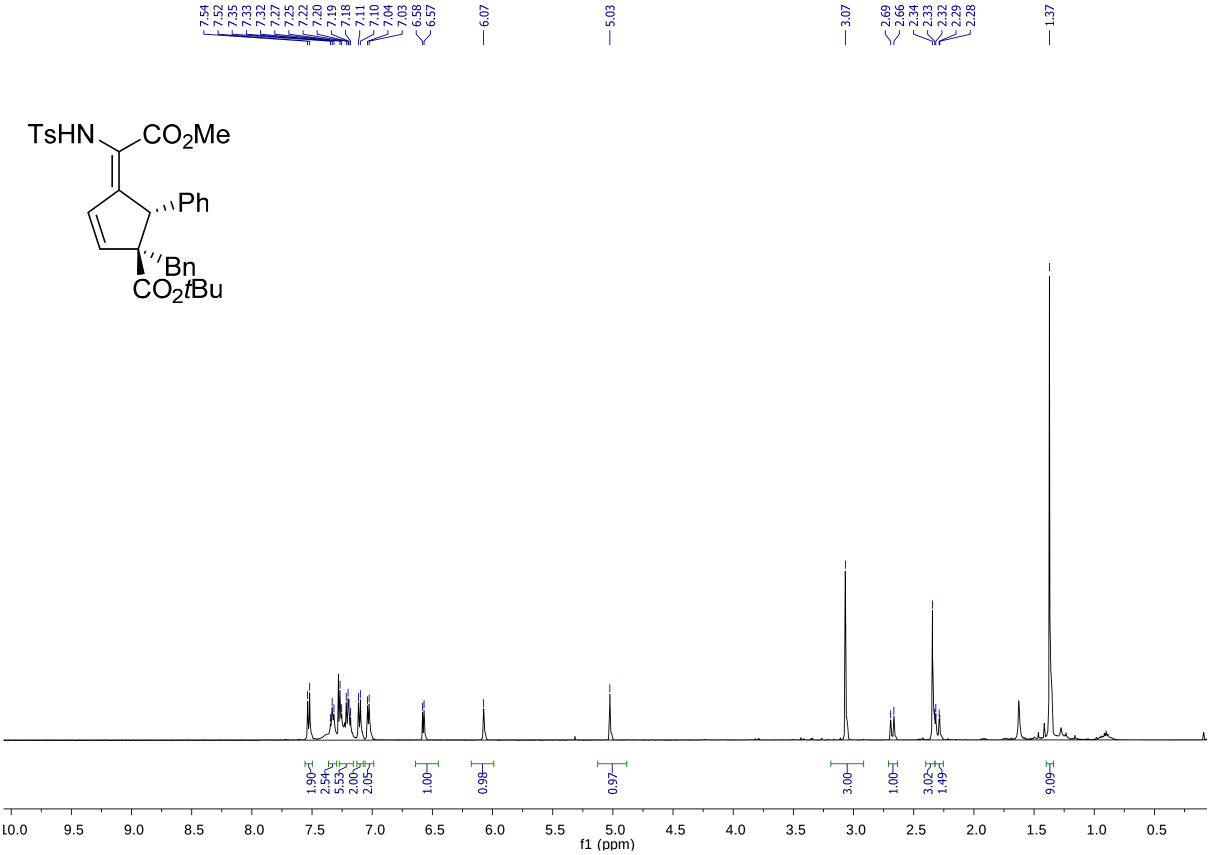

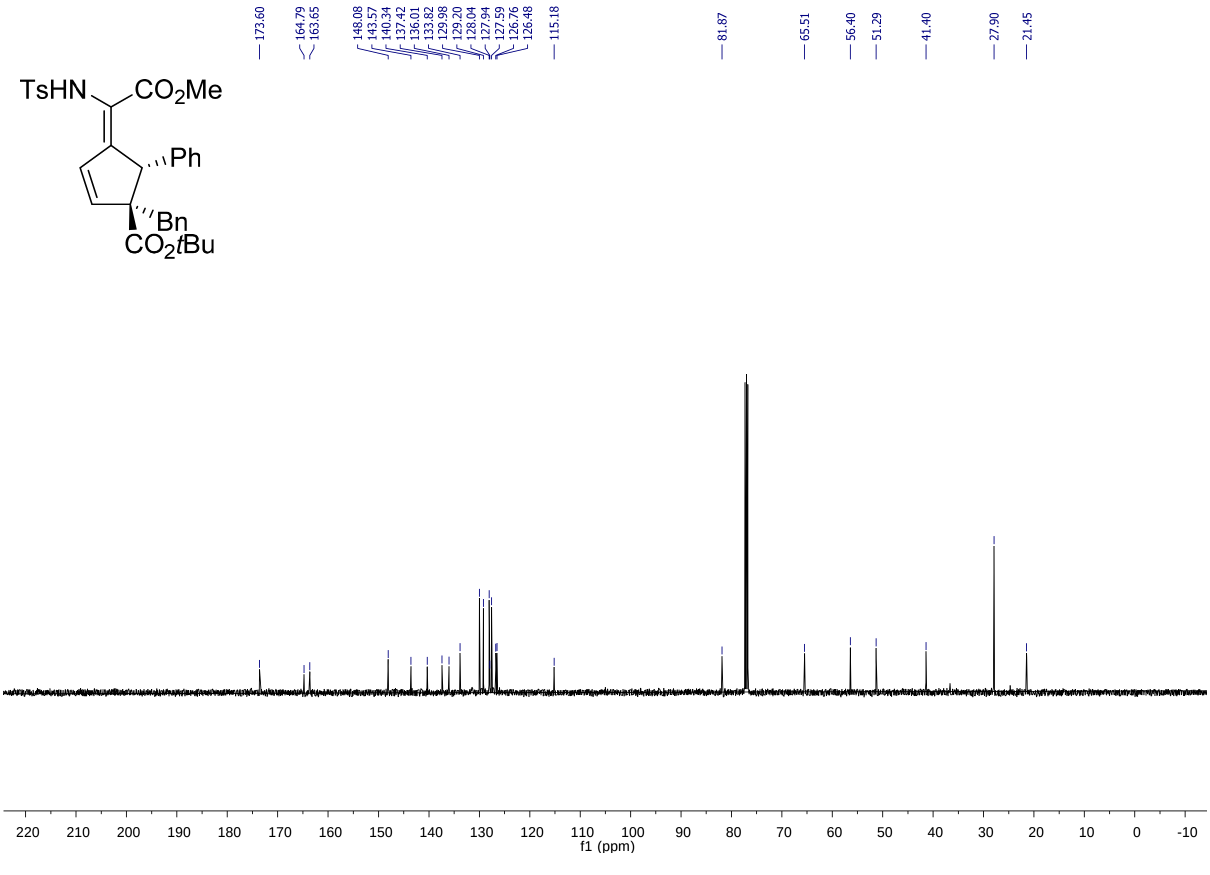

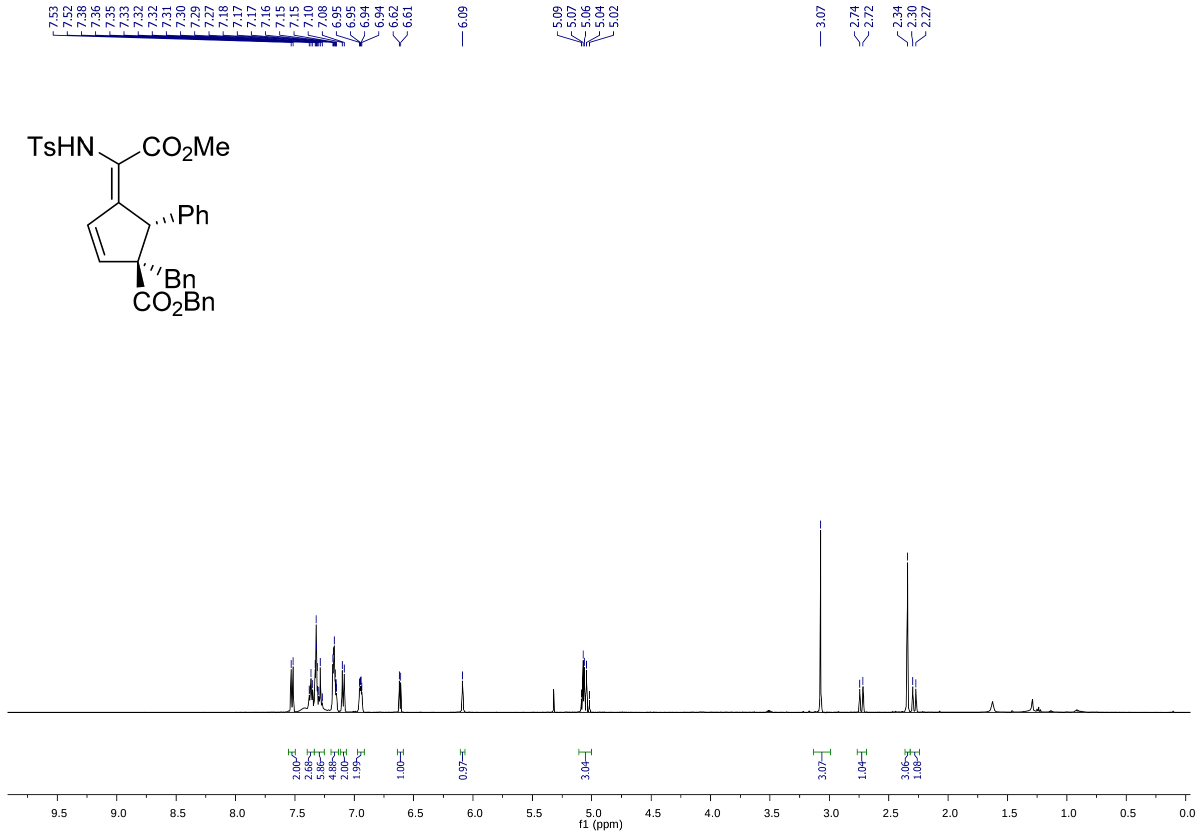
7.53 7.52 7.38 7.36 7.35 7.33 7.32 7.32 7.31 7.30 7.29 7.27 7.18 7.17 7.17 7.16 7.15 7.15 7.10 7.08 6.95 6.95 6.94 6.94 6.62 6.61
6.09
5.09 5.07 5.06 5.04 5.02
3.07
2.74 2.72
2.34 2.30 2.27
TsHN
CO2Me
Ph
Bn
CO2Bn
2.00 2.68 5.86 4.88 2.00 1.99 1.00 0.97 3.04 3.07 1.04 3.06 1.08
9.5 9.0 8.5 8.0 7.5 7.0 6.5 6.0 5.5 5.0 4.5 4.0 3.5 3.0 2.5 2.0 1.5 1.0 0.5 0.0
f1 (ppm)

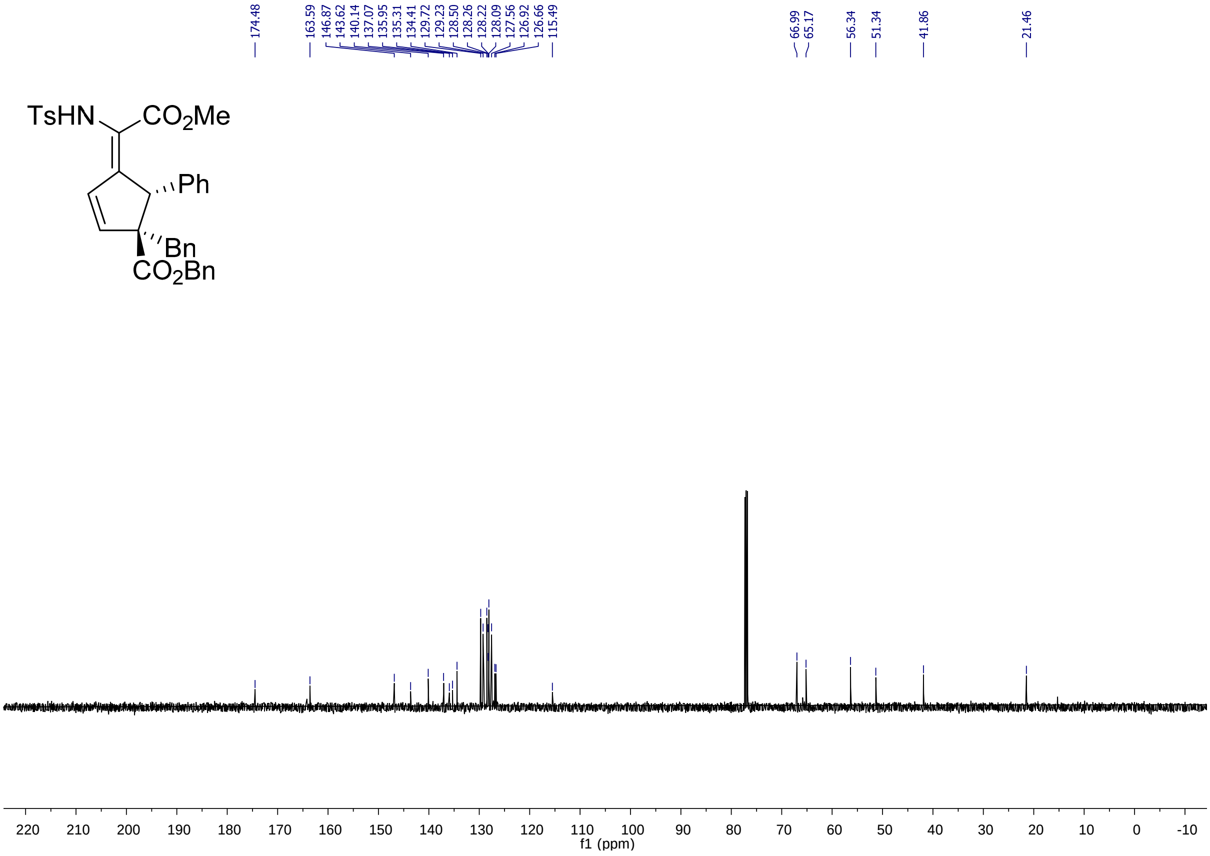
174.48
163.59 146.87 143.62 140.14 137.07 135.95 135.31 134.41 129.72 129.23 128.50 128.26 128.22 128.09 127.56 126.92 126.66 115.49
66.99 65.17
56.34
51.34
41.86
21.46
TsHN
CO2Me
Ph
Bn
CO2Bn
220 210 200 190 180 170 160 150 140 130 120 110 100 90 80 70 60 50 40 30 20 10 0 -10
f1 (ppm)

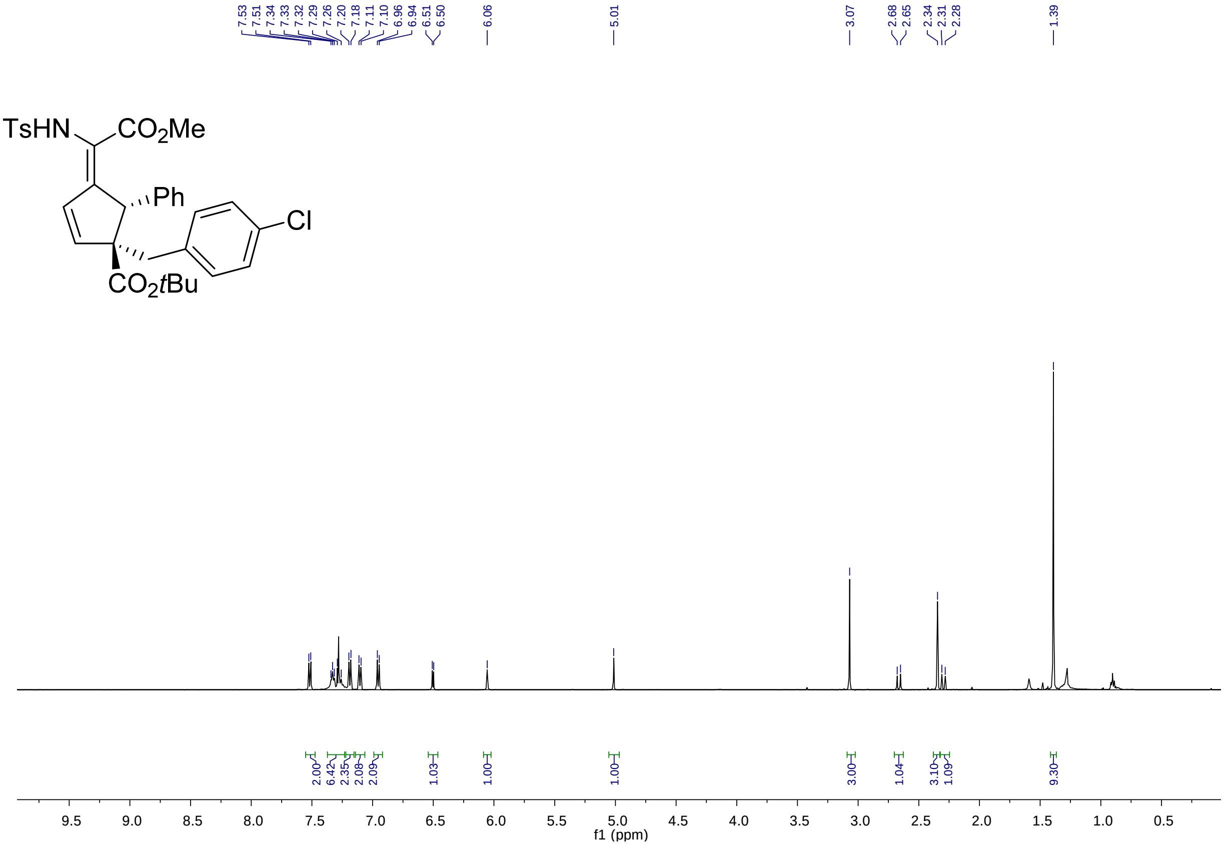
7.53 7.51 7.34 7.33 7.32 7.29 7.26 7.20 7.18 7.11 7.10 6.96 6.94 6.51 6.50
6.06
5.01
3.07
2.68 2.65
2.34 2.31 2.28
1.39
TsHN
CO2Me
Ph
Cl
CO2tBu
2.00 6.42 2.35 2.08 2.09
1.03
1.00
1.00
3.00
1.04
3.10 1.09
9.30
9.5 9.0 8.5 8.0 7.5 7.0 6.5 6.0 5.5 5.0 4.5 4.0 3.5 3.0 2.5 2.0 1.5 1.0 0.5
f1 (ppm)

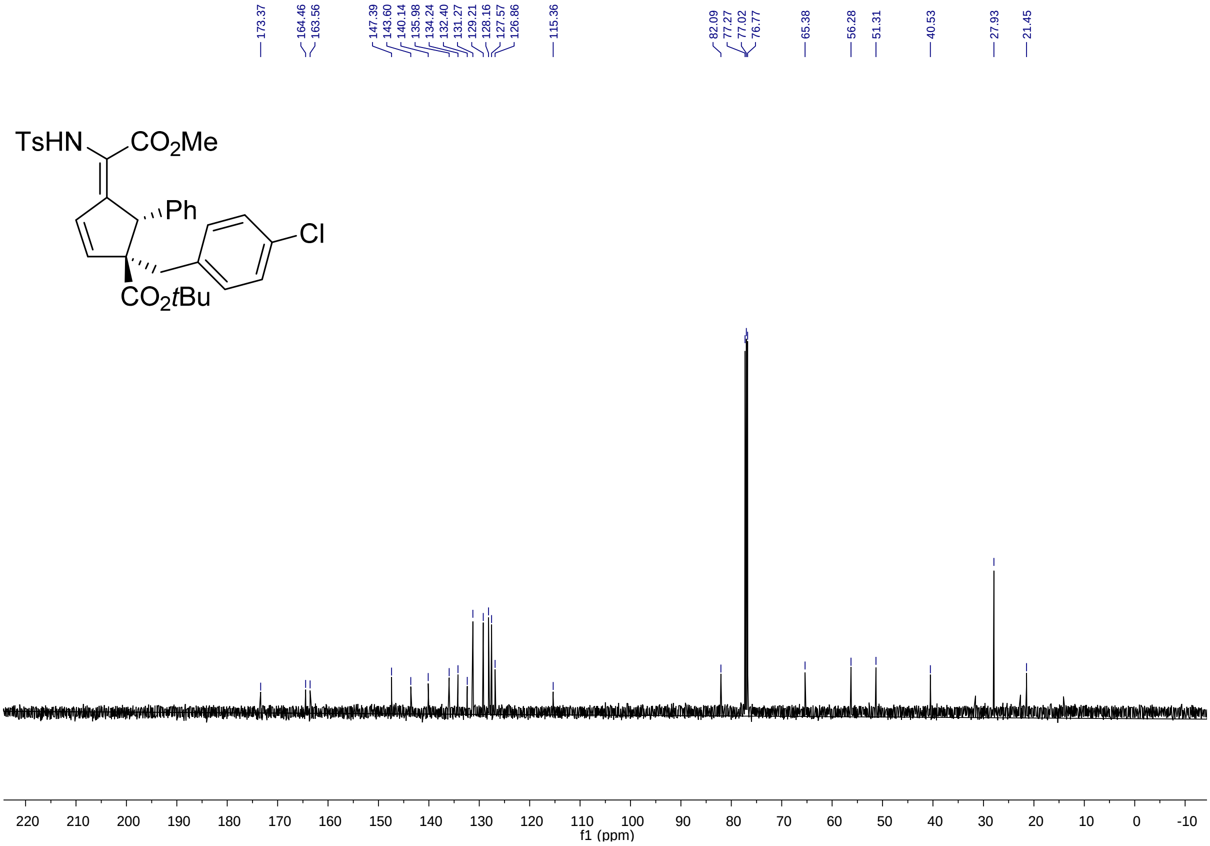
173.37
164.46 163.56
147.39 143.60 140.14 135.98 134.24 132.40 131.27 129.21 128.16 127.57 126.86
115.36
82.09 77.27 77.02 76.77
65.38
56.28
51.31
40.53
27.93
21.45
TsHN
CO2Me
Ph
Cl
CO2tBu
220 210 200 190 180 170 160 150 140 130 120 110 100 90 80 70 60 50 40 30 20 10 0 -10
f1 (ppm)

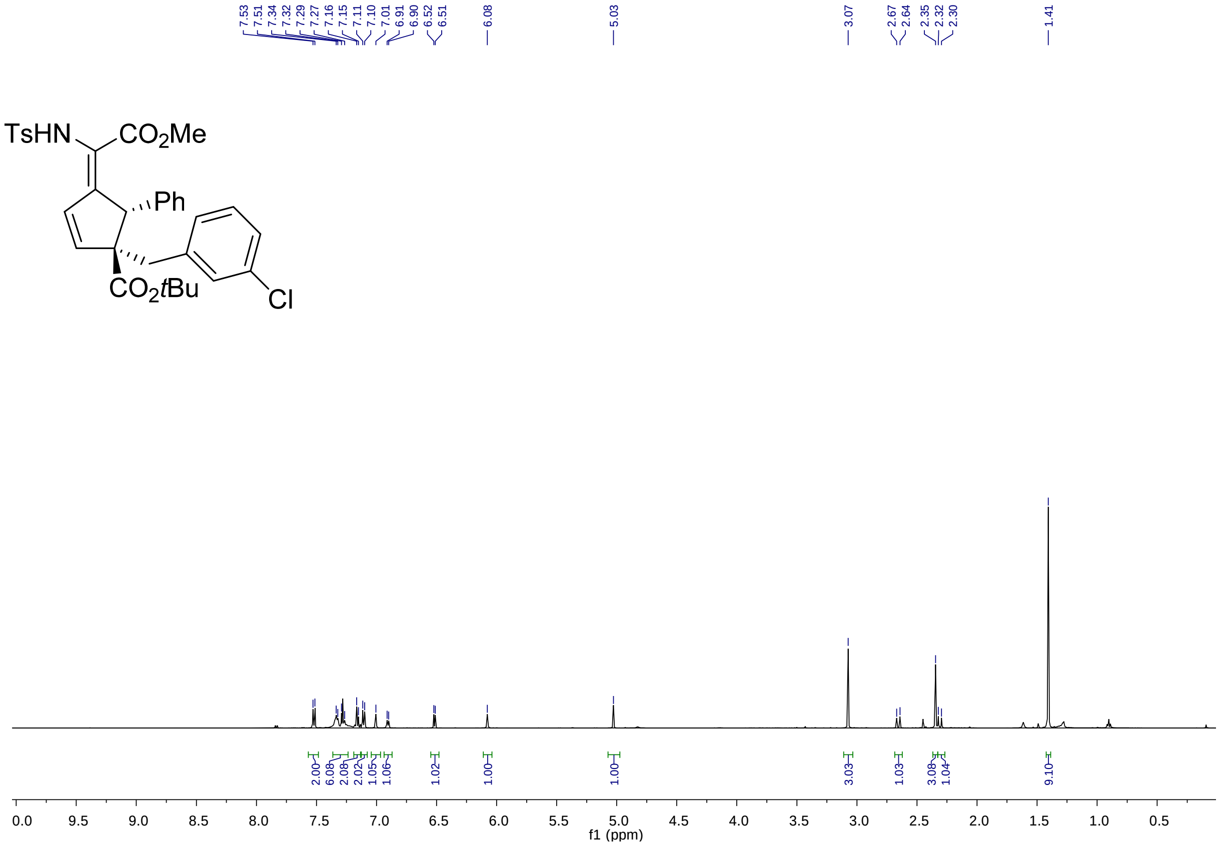
7.53 7.51 7.34 7.32 7.29 7.27 7.16 7.15 7.11 7.10 7.01 6.91 6.90 6.52 6.51
6.08
5.03
3.07
2.67 2.64 2.35 2.32 2.30
1.41
TsHN
CO2Me
Ph
CO2tBu
Cl
2.00 6.08 2.08 2.02 1.05 1.06
1.02
1.00
1.00
3.03
1.03
3.08 1.04
9.10
f1 (ppm)

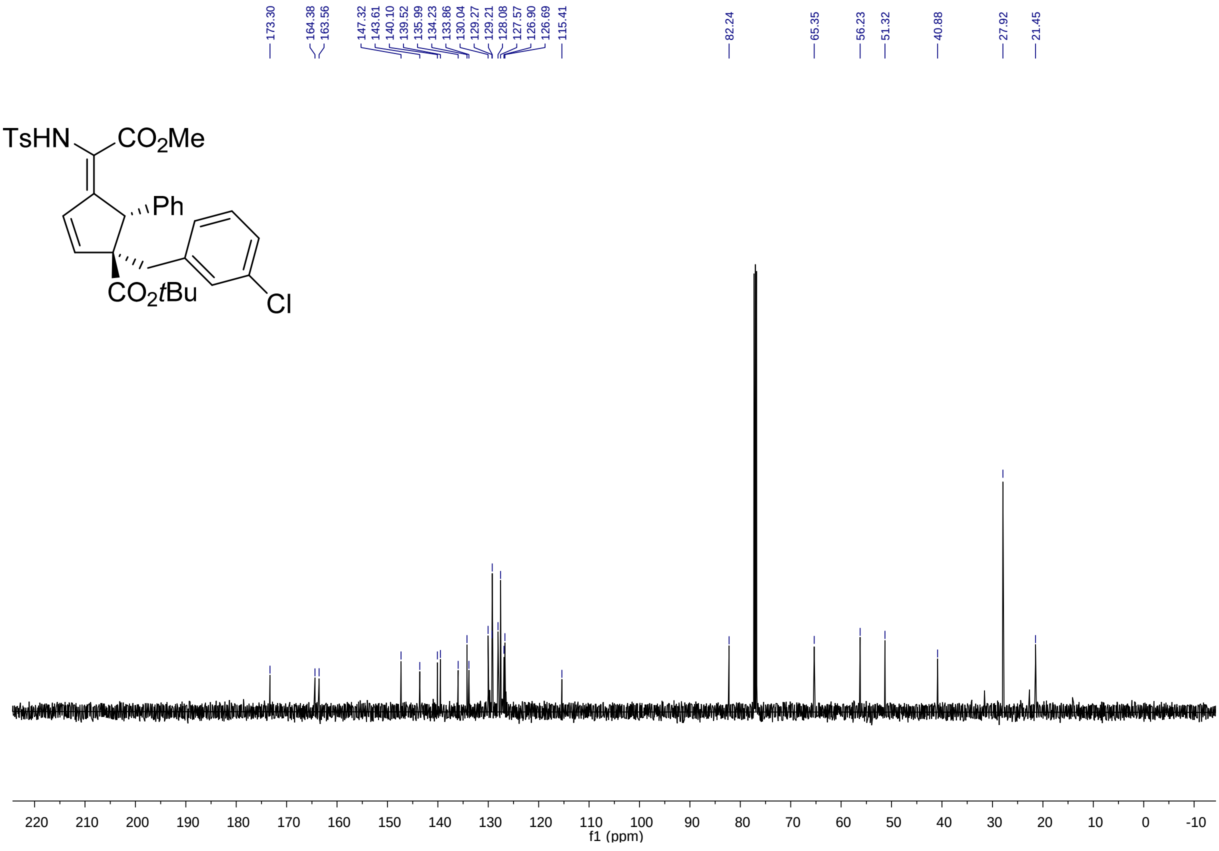
173.30
164.38 163.56
147.32 143.61 140.10 139.52 135.99 134.23 133.86 130.04 129.27 129.21 128.08 127.57 126.90 126.69 115.41
82.24
65.35
56.23
51.32
40.88
27.92
21.45
TsHN
CO2Me
Ph
CO2tBu
Cl
f1 (ppm)

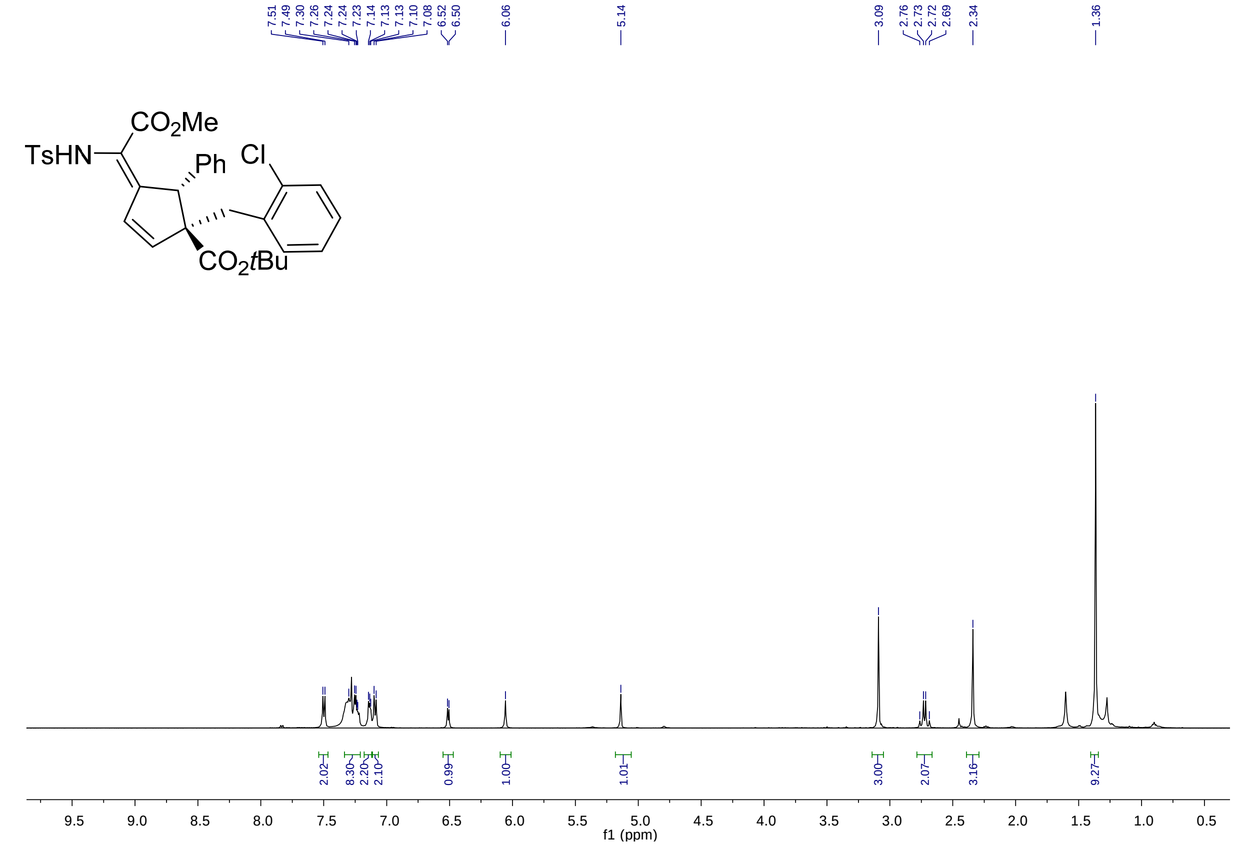
CO2Me
TsHN
Ph
Cl
CO2tBu
7.51 7.49 7.30 7.26 7.24 7.24 7.23 7.14 7.13 7.13 7.10 7.08 6.52 6.50
6.06
5.14
3.09 2.76 2.73 2.72 2.69 2.34
1.36
2.02 8.30 2.20 2.10 0.99 1.00 1.01 3.00 2.07 3.16 9.27
f1 (ppm)

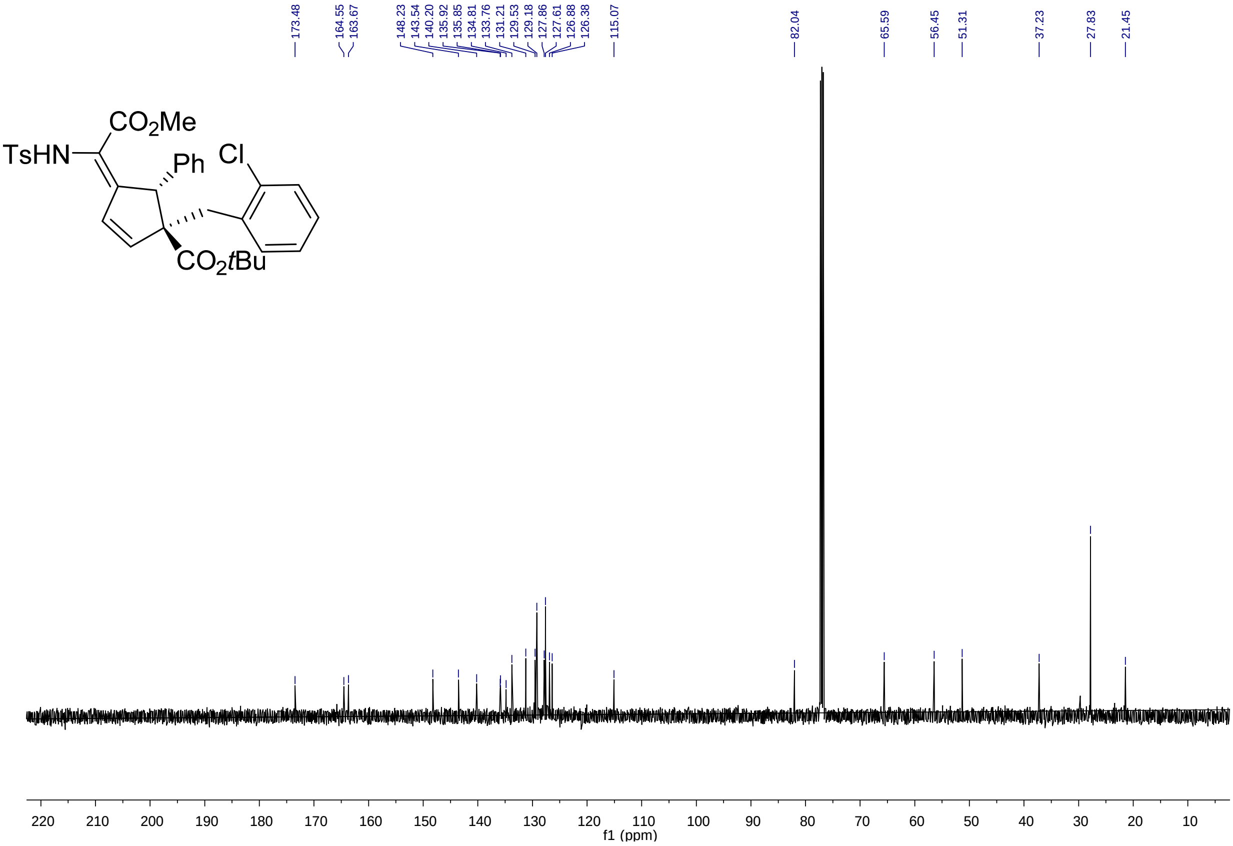
CO2Me
TsHN
Ph
Cl
CO2tBu
173.48
164.55 163.67
148.23 143.54 140.20 135.92 135.85 134.81 133.76 131.21 129.53 129.18 127.86 127.61 126.88 126.38
115.07
82.04
65.59
56.45 51.31
37.23
27.83
21.45
f1 (ppm)

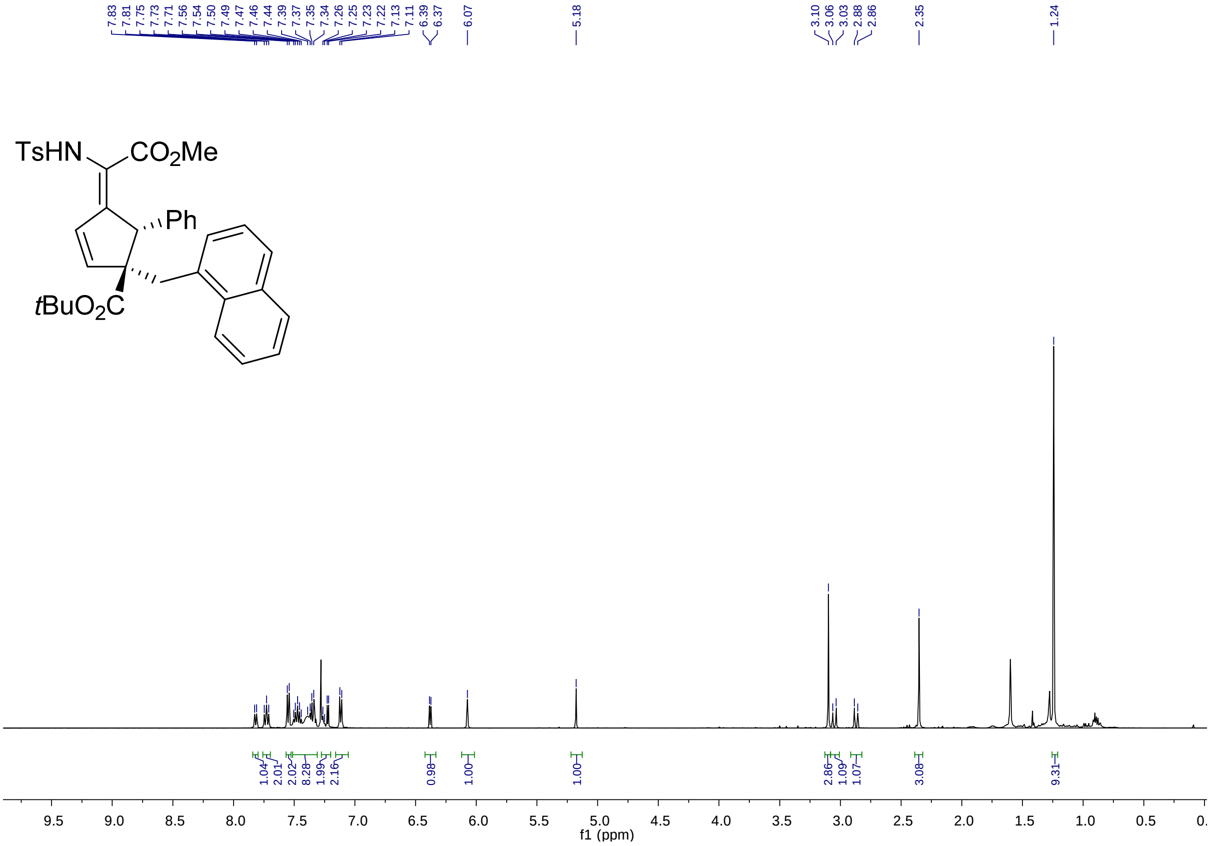
7.83 7.81 7.75 7.73 7.71 7.56 7.54 7.50 7.49 7.47 7.46 7.44 7.39 7.37 7.35 7.34 7.26 7.25 7.23 7.22 7.13 7.11 6.39 6.37 6.07 5.18 3.10 3.06 3.03 2.88 2.86 2.35 1.24
TsHN
CO2Me
Ph
tBuO2C
1.04 2.01 2.02 8.28 1.99 2.16 0.98 1.00 1.00 2.86 1.09 1.07 3.08 9.31
f1 (ppm)

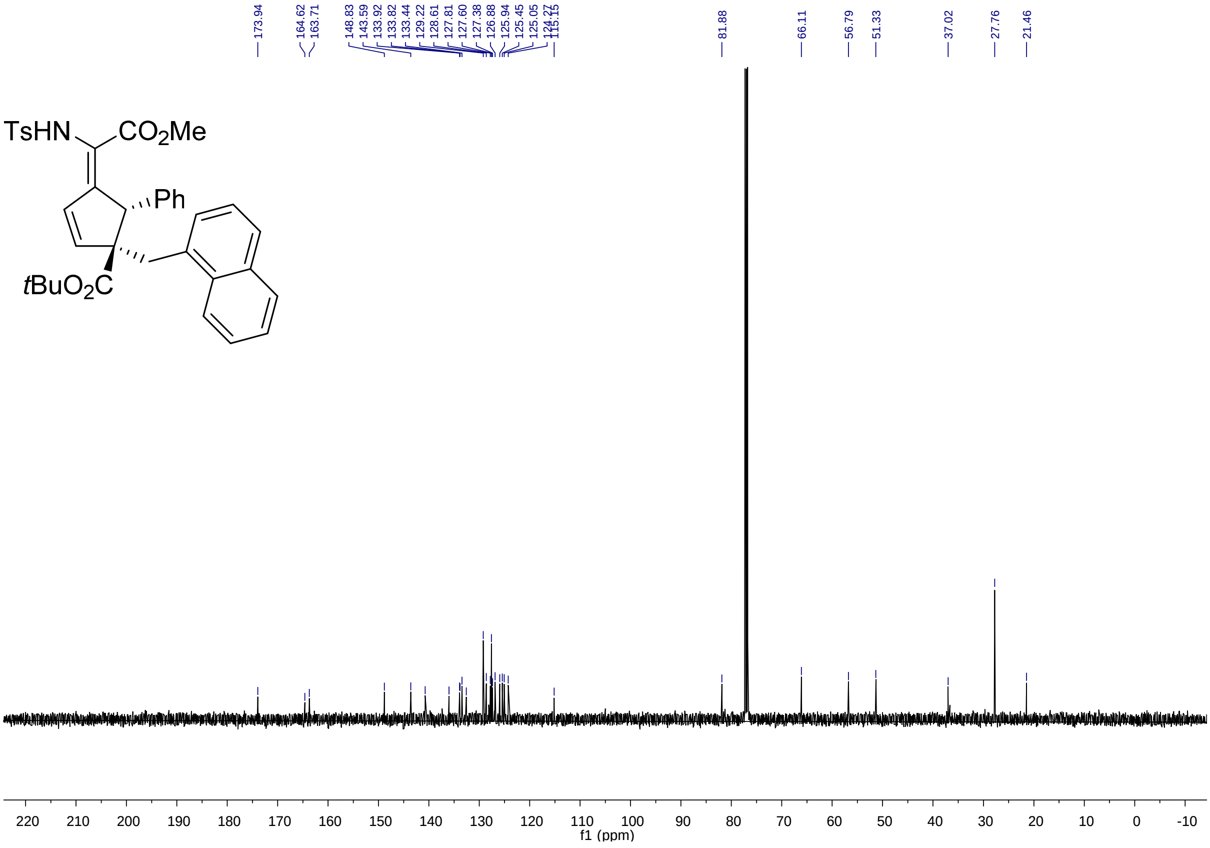
173.94 164.62 163.71 148.83 143.59 133.92 133.82 133.44 129.22 128.61 127.81 127.60 127.38 126.88 125.94 125.45 125.05 124.27 115.15 81.88 66.11 56.79 51.33 37.02 27.76 21.46
TsHN
CO2Me
Ph
tBuO2C
f1 (ppm)

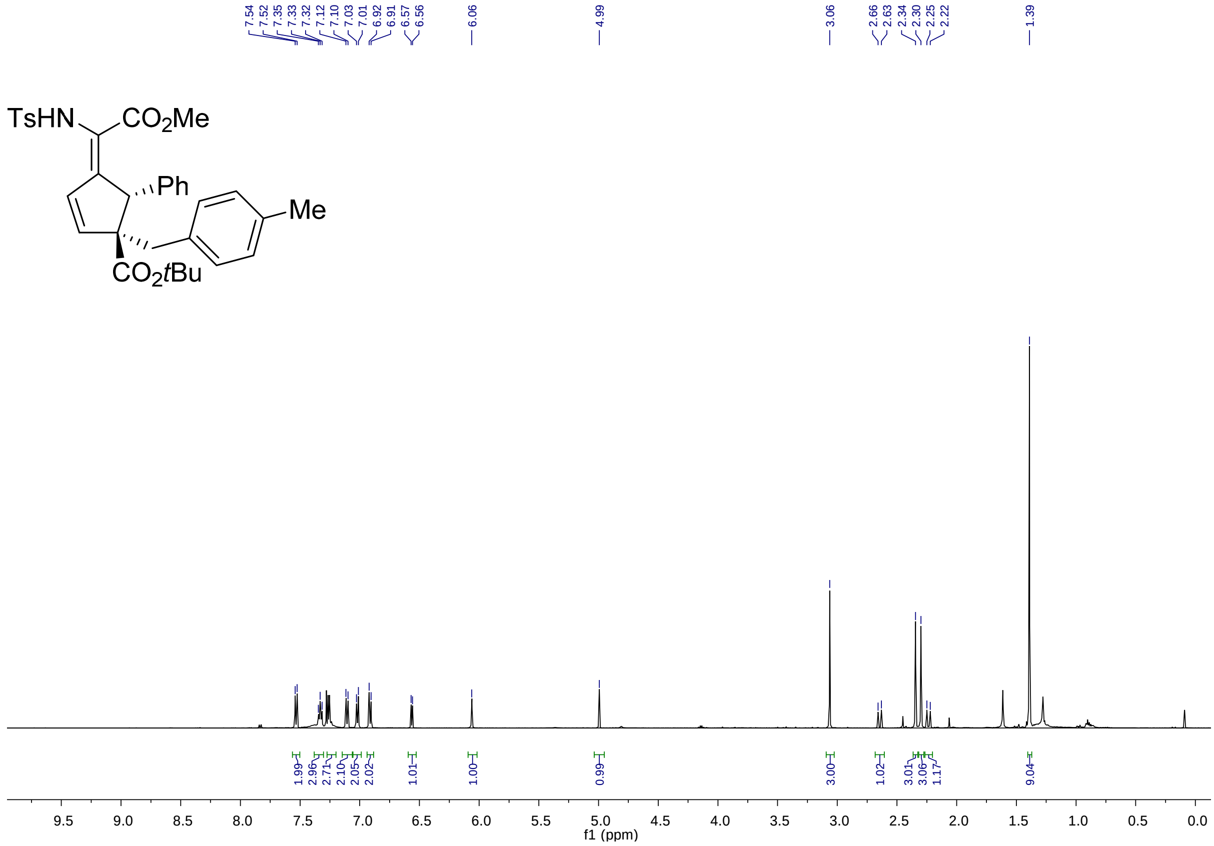
7.54 7.52 7.35 7.33 7.32 7.12 7.10 7.03 7.01 6.92 6.91 6.57 6.56
6.06
4.99
3.06
2.66 2.63 2.34 2.30 2.25 2.22
1.39
TsHN
CO2Me
Ph
Me
CO2tBu
1.99 2.96 2.71 2.10 2.05 2.02
1.01
1.00
0.99
3.00
1.02
3.01 3.06 1.17
9.04
9.5 9.0 8.5 8.0 7.5 7.0 6.5 6.0 5.5 5.0 4.5 4.0 3.5 3.0 2.5 2.0 1.5 1.0 0.5 0.0
f1 (ppm)

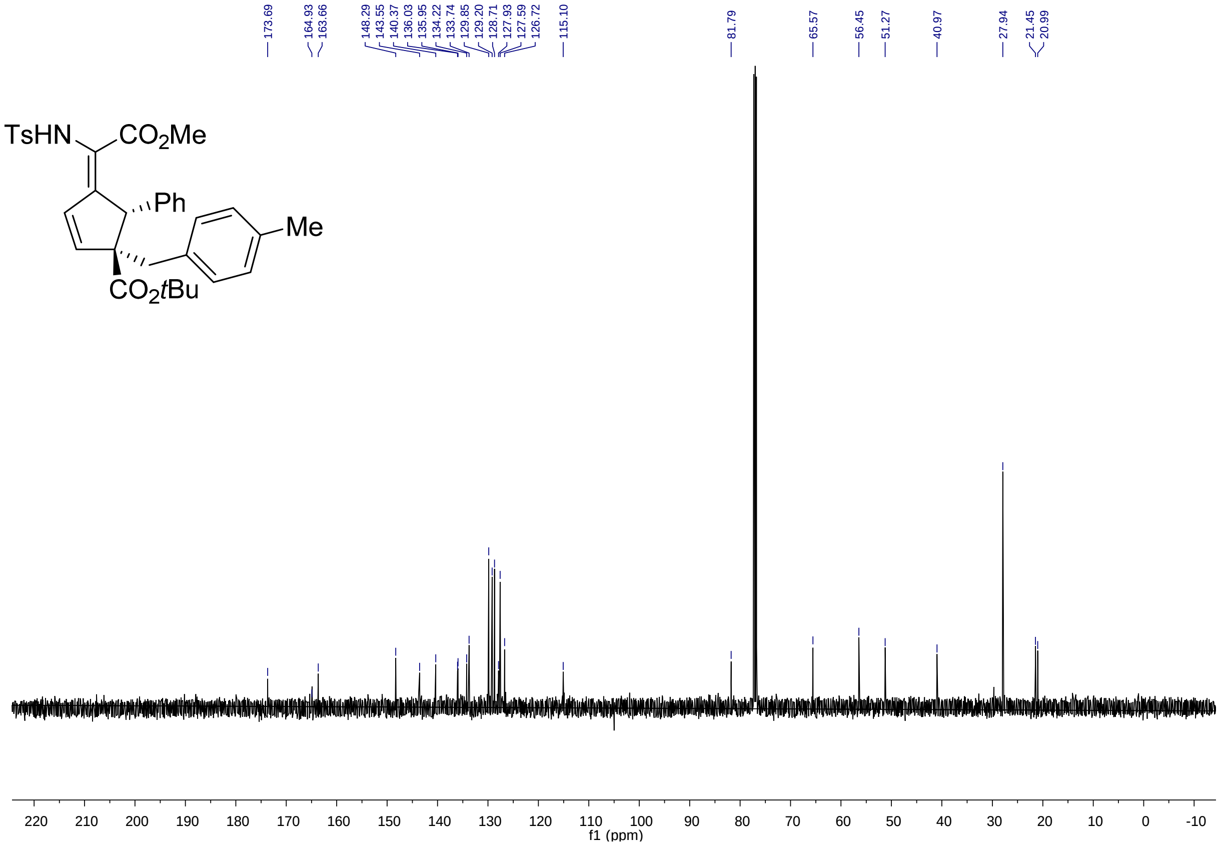
173.69
164.93 163.66
148.29 143.55 140.37 136.03 135.95 134.22 133.74 129.85 129.20 128.71 127.93 127.59 126.72
115.10
81.79
65.57
56.45
51.27
40.97
27.94
21.45 20.99
TsHN
CO2Me
Ph
Me
CO2tBu
220 210 200 190 180 170 160 150 140 130 120 110 100 90 80 70 60 50 40 30 20 10 0 -10
f1 (ppm)

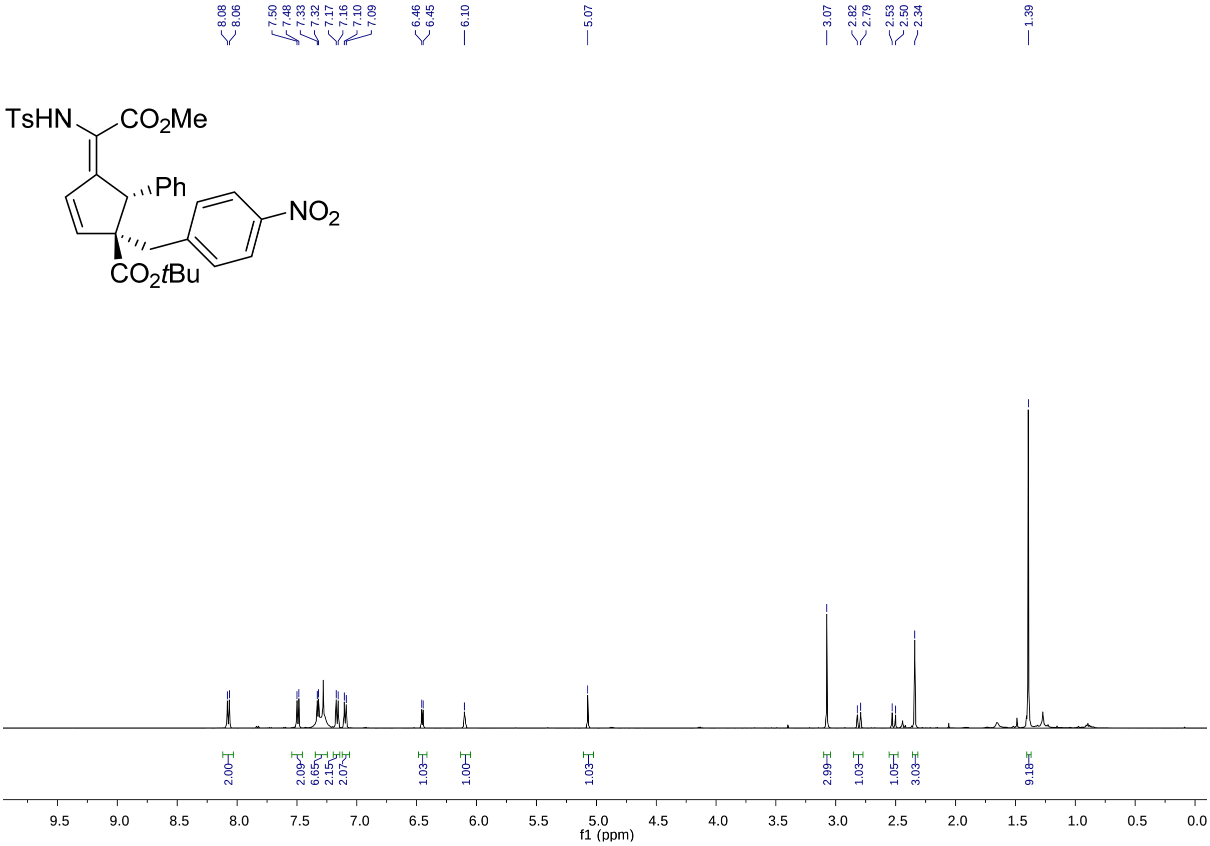
8.08 8.06 7.50 7.48 7.33 7.32 7.17 7.16 7.10 7.09 6.46 6.45 6.10 5.07 3.07 2.82 2.79 2.53 2.50 2.34 1.39
TsHN
CO2Me
Ph
NO2
CO2tBu
2.00 2.09 6.65 2.15 2.07 1.03 1.00 1.03 2.99 1.03 1.05 3.03 9.18
f1 (ppm)

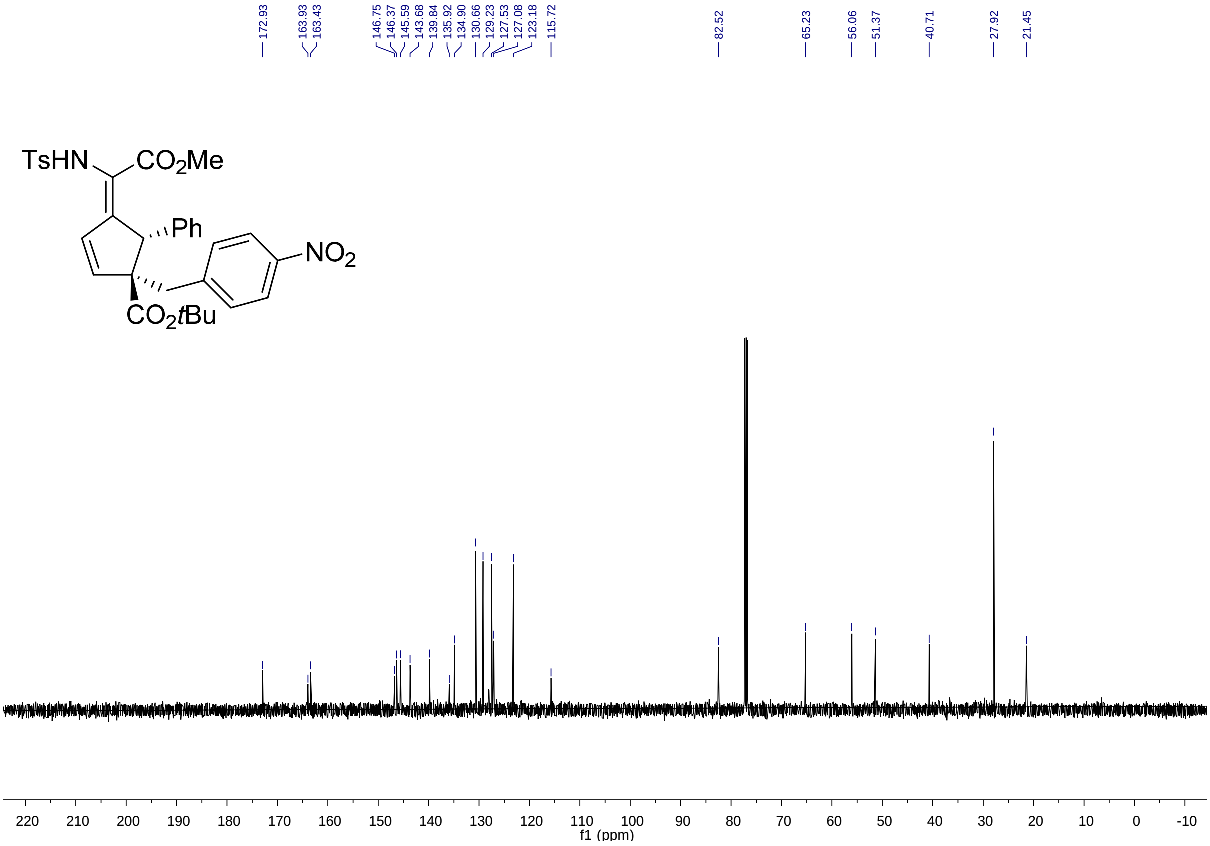
172.93 163.93 163.43 146.75 146.37 145.59 143.68 139.84 135.92 134.90 130.66 129.23 127.53 127.08 123.18 115.72 82.52 65.23 56.06 51.37 40.71 27.92 21.45
TsHN
CO2Me
Ph
NO2
CO2tBu
f1 (ppm)

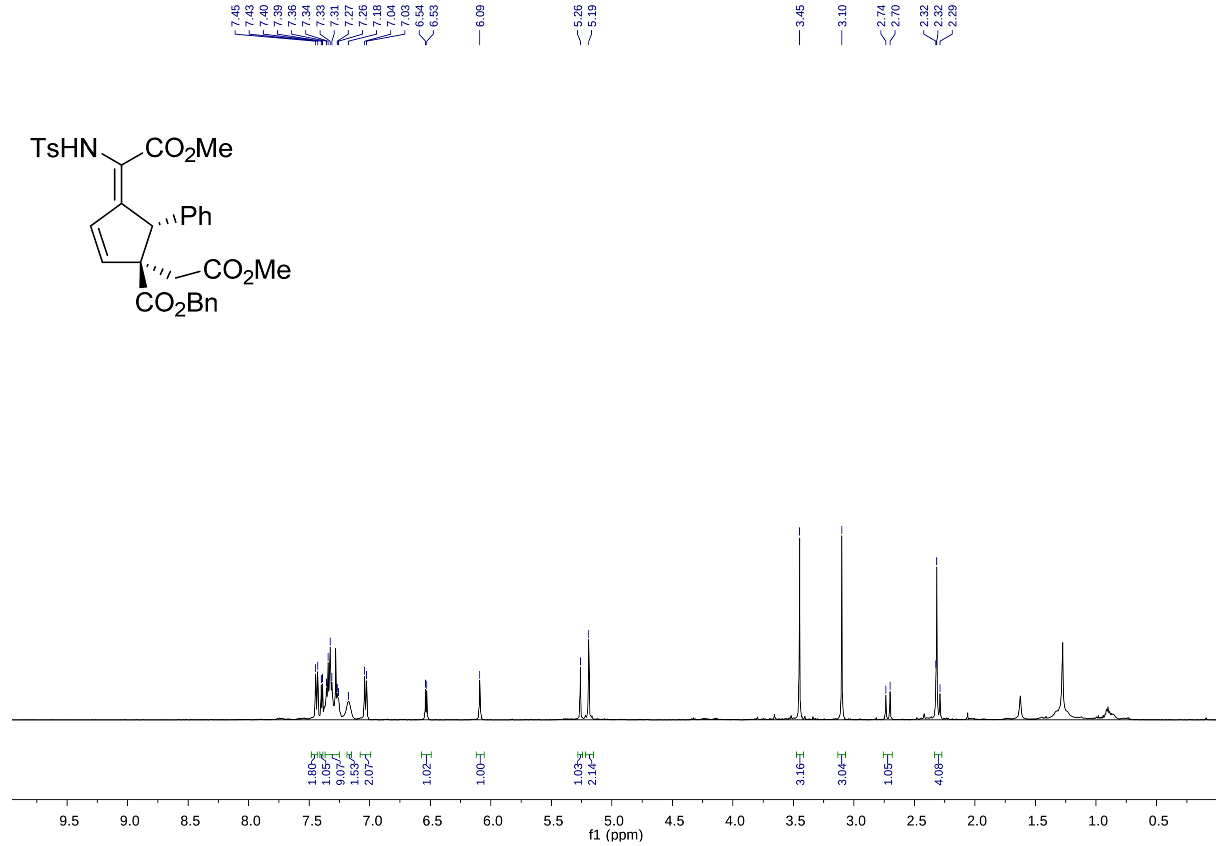
7.45
7.43
7.40
7.39
7.36
7.34
7.33
7.31
7.27
7.26
7.18
7.04
7.03
6.54
6.53
6.09
5.26
5.19
3.45
3.10
2.74
2.70
2.32
2.32
2.29
TsHN
CO2Me
Ph
CO2Me
CO2Bn
1.80
1.05
9.07
1.53
2.07
1.02
1.00
1.03
2.14
3.16
3.04
1.05
4.08
f1 (ppm)

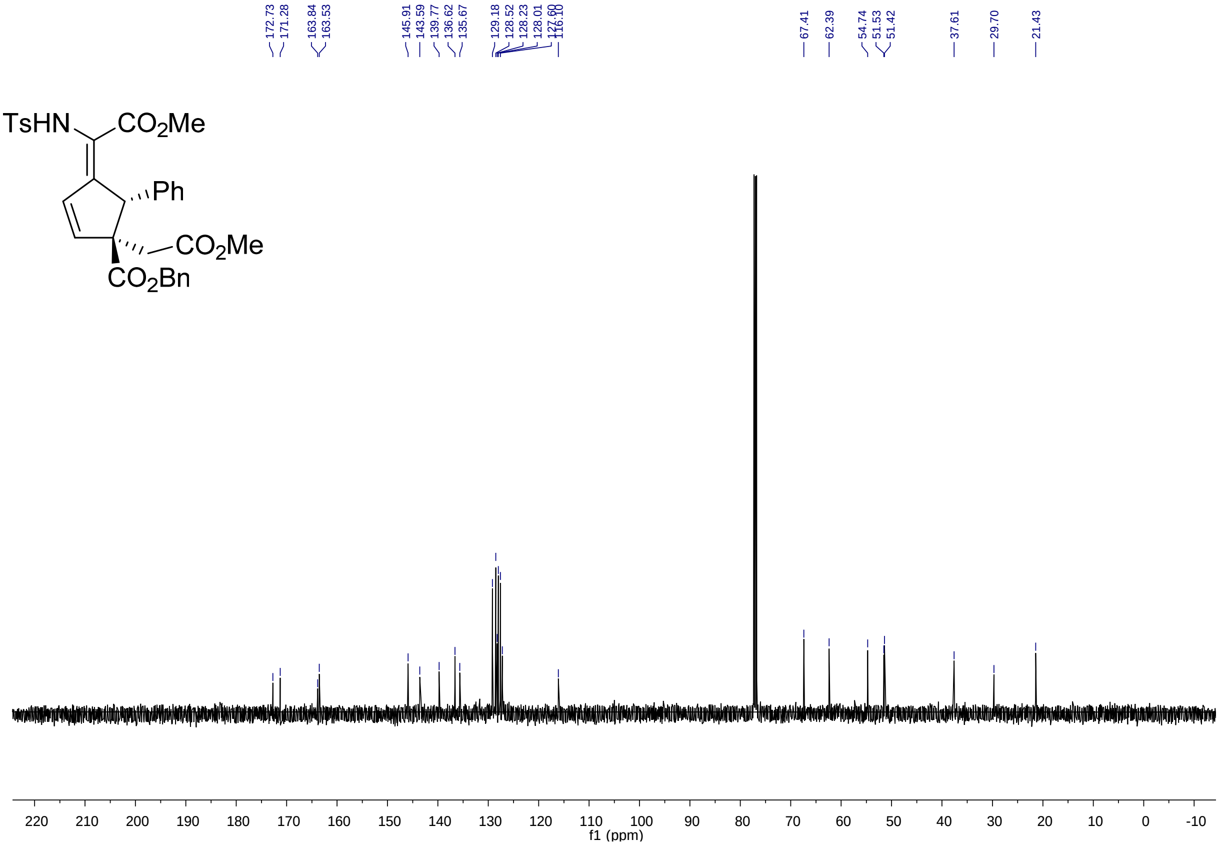
172.73
171.28
163.84
163.53
145.91
143.59
139.77
136.62
135.67
129.18
128.52
128.23
128.01
116.10
67.41
62.39
54.74
51.53
51.42
37.61
29.70
21.43
TsHN
CO2Me
Ph
CO2Me
CO2Bn
f1 (ppm)

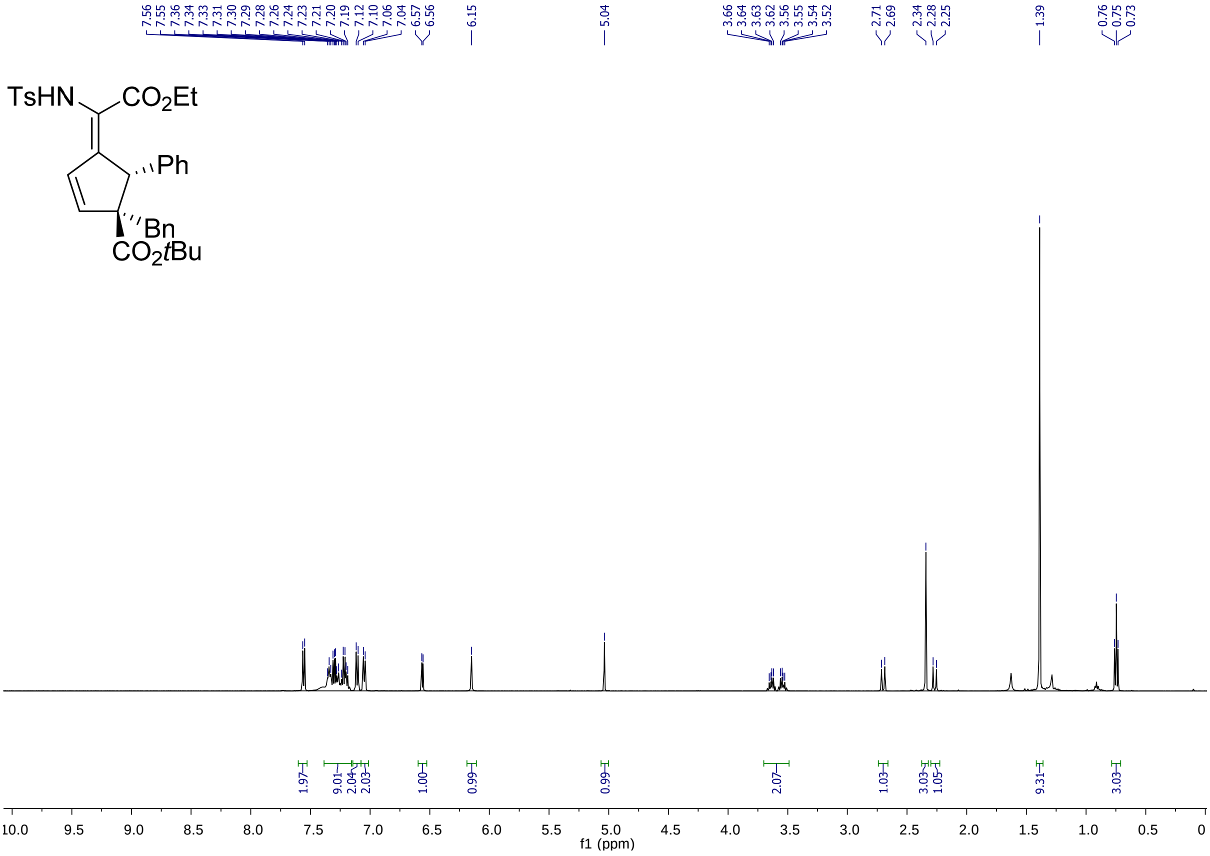

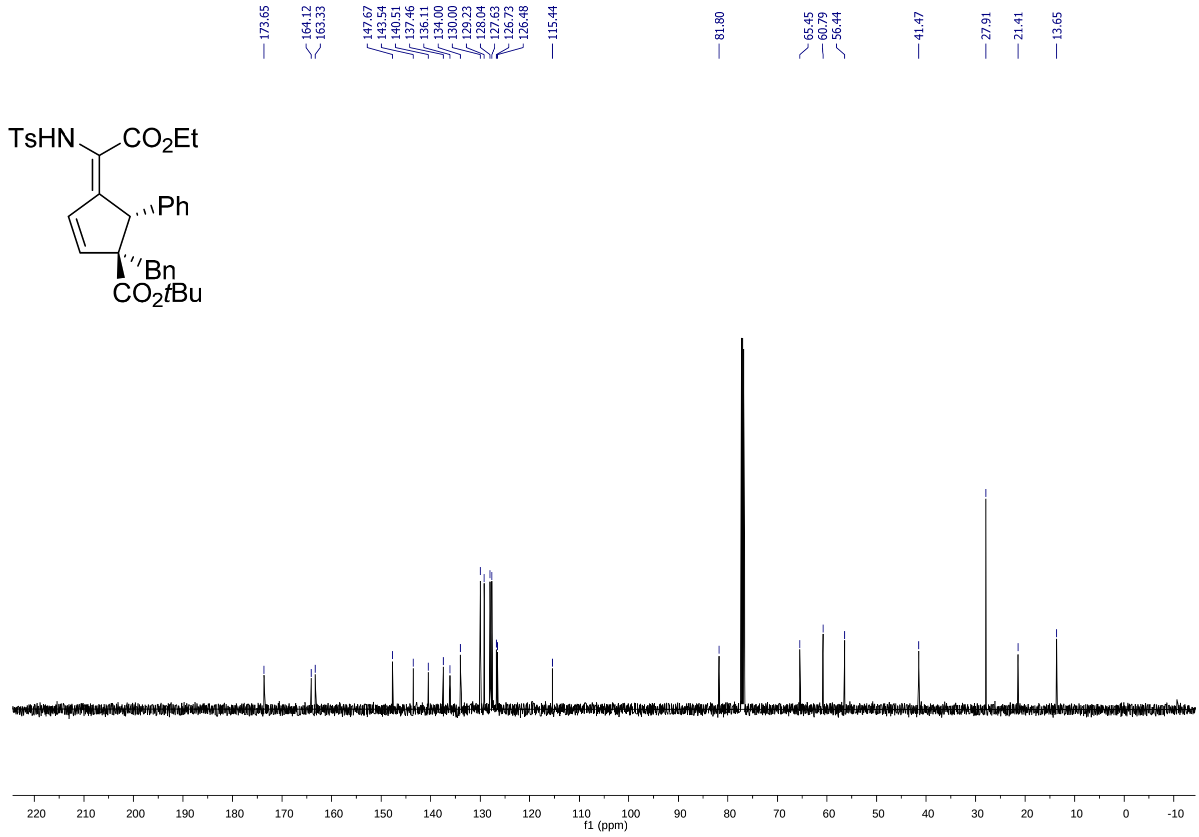

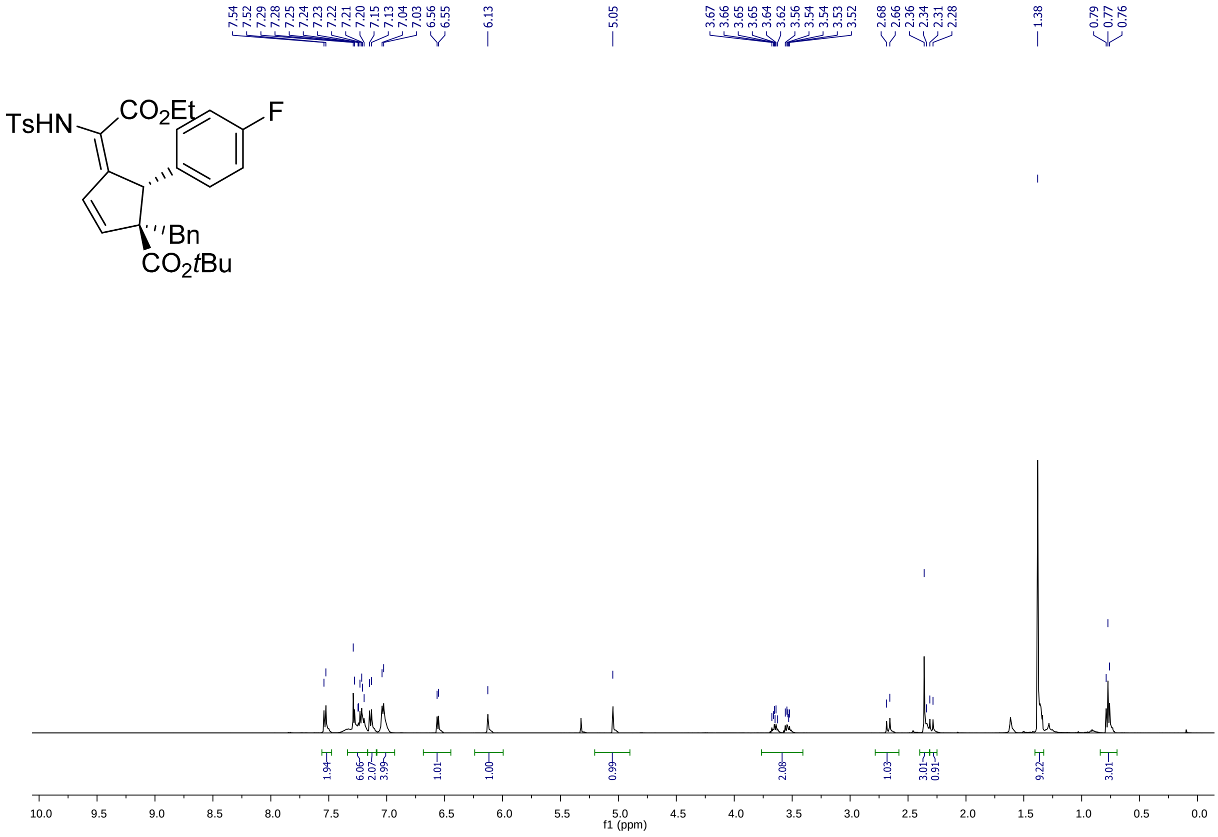
7.54 7.52 7.29 7.28 7.25 7.24 7.23 7.22 7.21 7.20 7.15 7.13 7.04 7.03 6.56 6.55 6.13 5.05 3.67 3.66 3.65 3.65 3.64 3.62 3.56 3.54 3.54 3.53 3.52 2.68 2.66 2.36 2.34 2.31 2.28 1.38 0.79 0.77 0.76
TsHN
CO_2Et
F
Bn
CO_2tBu
1.94 6.06 2.07 3.99 1.01 1.00 0.99 2.08 1.03 3.01 0.91 9.22 3.01
f1 (ppm)

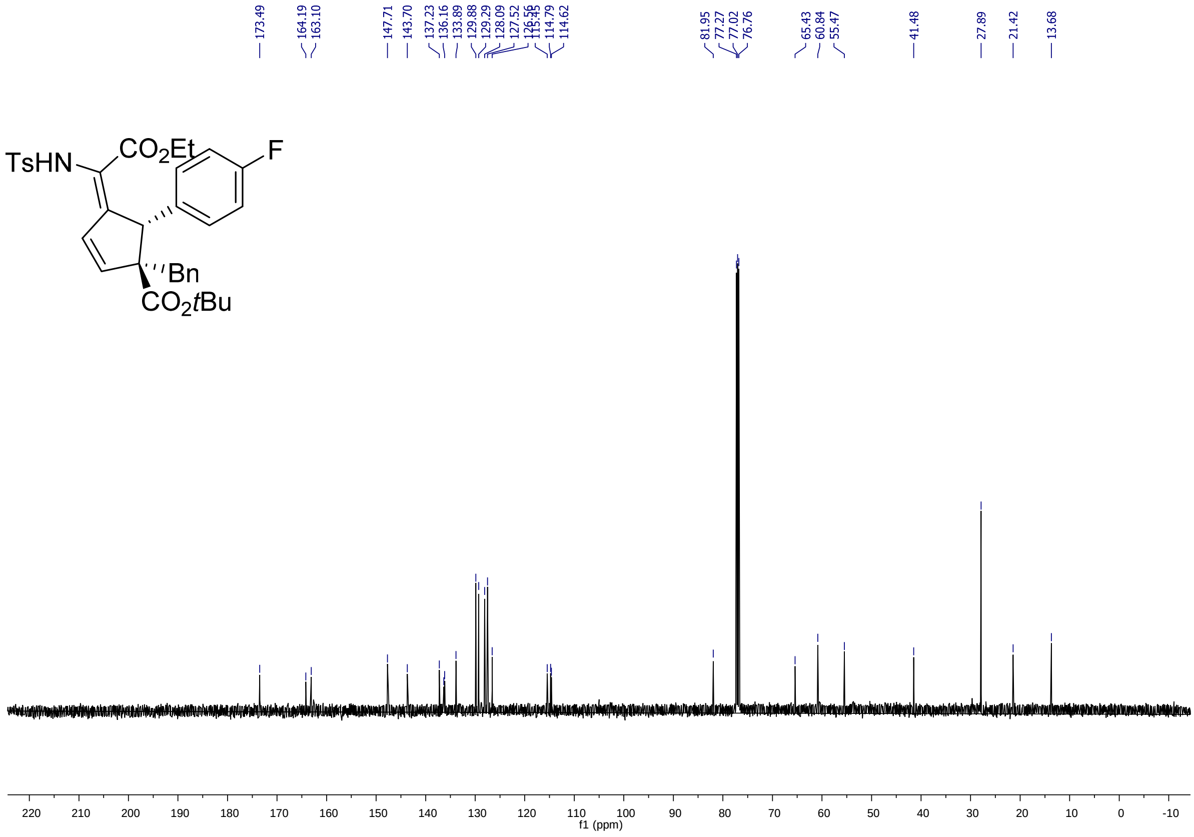
173.49 164.19 163.10 147.71 143.70 137.23 136.16 133.89 129.88 129.29 128.09 127.52 126.56 115.45 114.79 114.62 81.95 77.27 77.02 76.76 65.43 60.84 55.47 41.48 27.89 21.42 13.68
TsHN
CO_2Et
F
Bn
CO_2tBu
f1 (ppm)

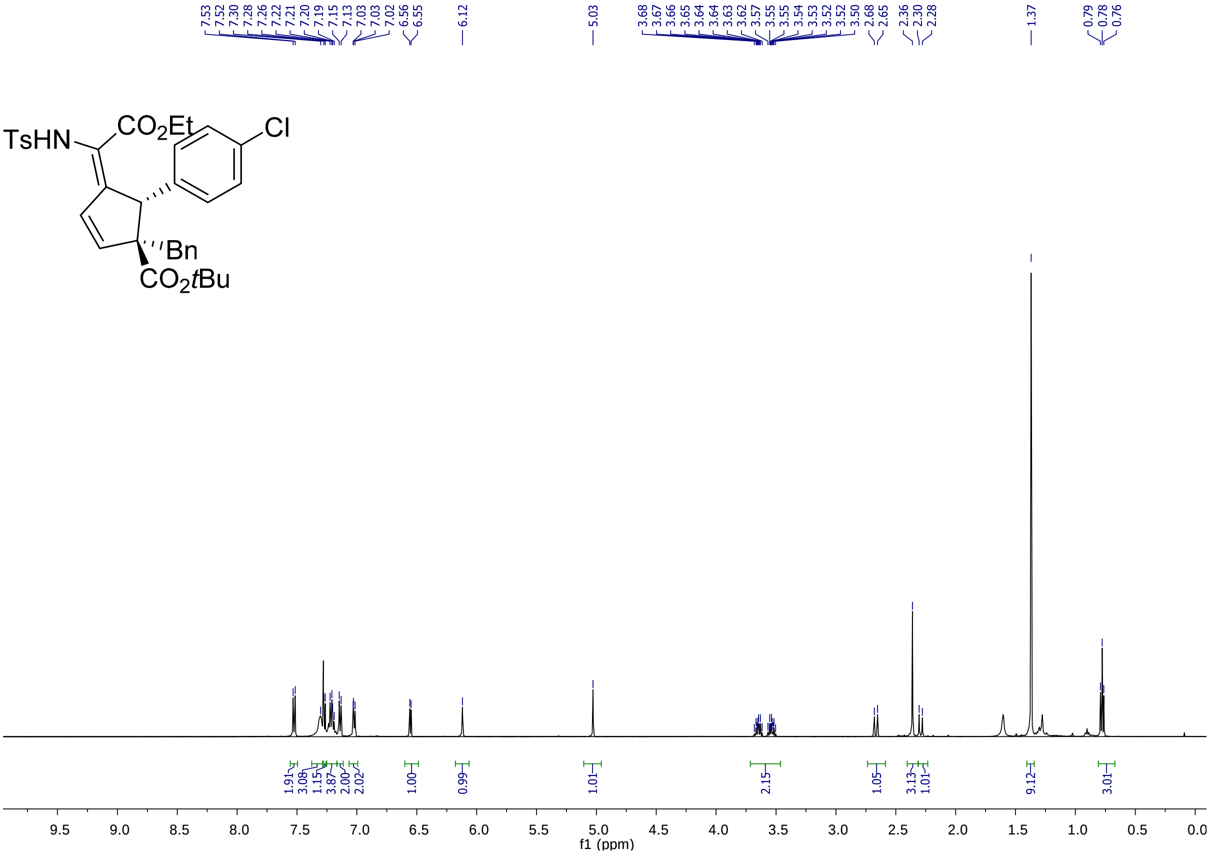
7.53
7.52
7.30
7.28
7.26
7.22
7.21
7.20
7.19
7.15
7.13
7.03
7.03
7.02
6.56
6.55
6.12
5.03
3.68
3.67
3.66
3.65
3.64
3.64
3.63
3.62
3.57
3.55
3.55
3.54
3.53
3.52
3.52
3.50
2.68
2.65
2.36
2.30
2.28
1.37
0.79
0.78
0.76
TsHN
CO2Et
Cl
Bn
CO2tBu
1.91
3.08
1.15
3.87
2.00
2.02
1.00
0.99
1.01
2.15
1.05
3.13
1.01
9.12
3.01
9.5 9.0 8.5 8.0 7.5 7.0 6.5 6.0 5.5 5.0 4.5 4.0 3.5 3.0 2.5 2.0 1.5 1.0 0.5 0.0
f1 (ppm)

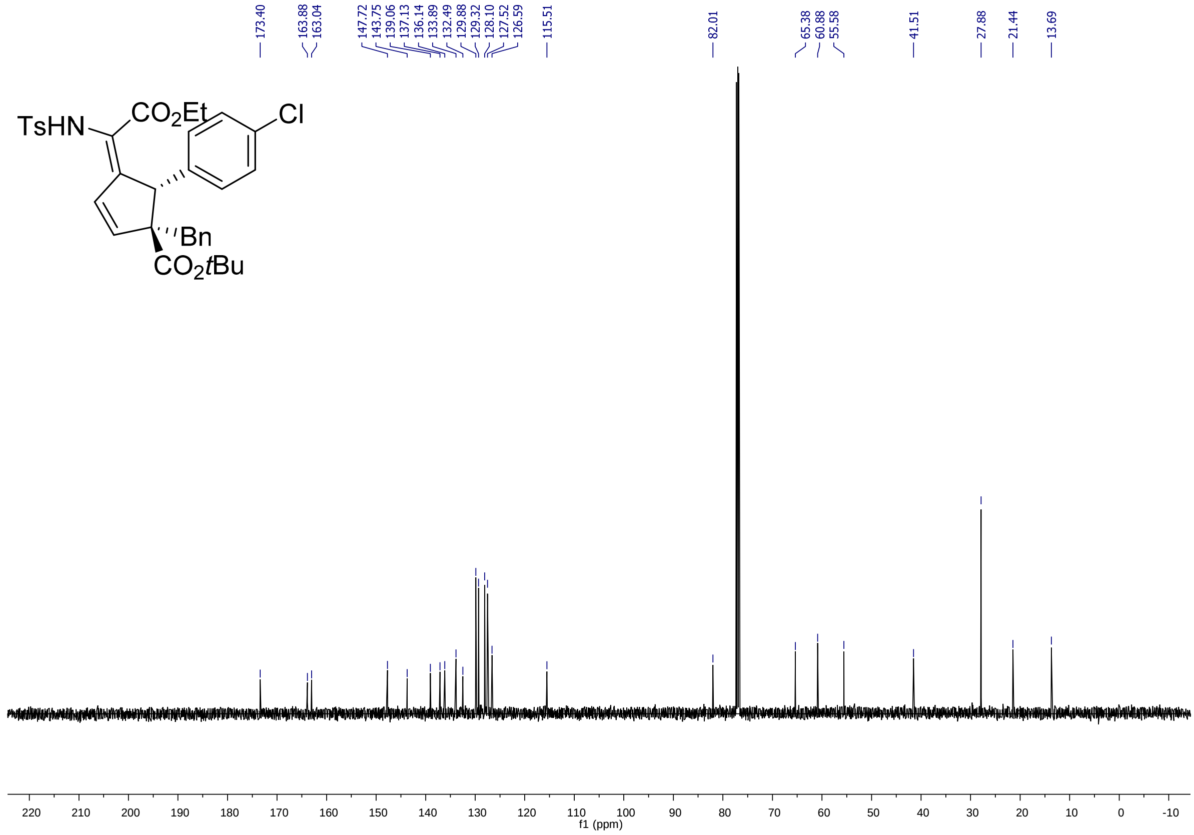
173.40
163.88
163.04
147.72
143.75
139.06
137.13
136.14
133.89
132.49
129.88
129.32
128.10
127.52
126.59
115.51
82.01
65.38
60.88
55.58
41.51
27.88
21.44
13.69
TsHN
CO2Et
Cl
Bn
CO2tBu
220 210 200 190 180 170 160 150 140 130 120 110 100 90 80 70 60 50 40 30 20 10 0 -10
f1 (ppm)

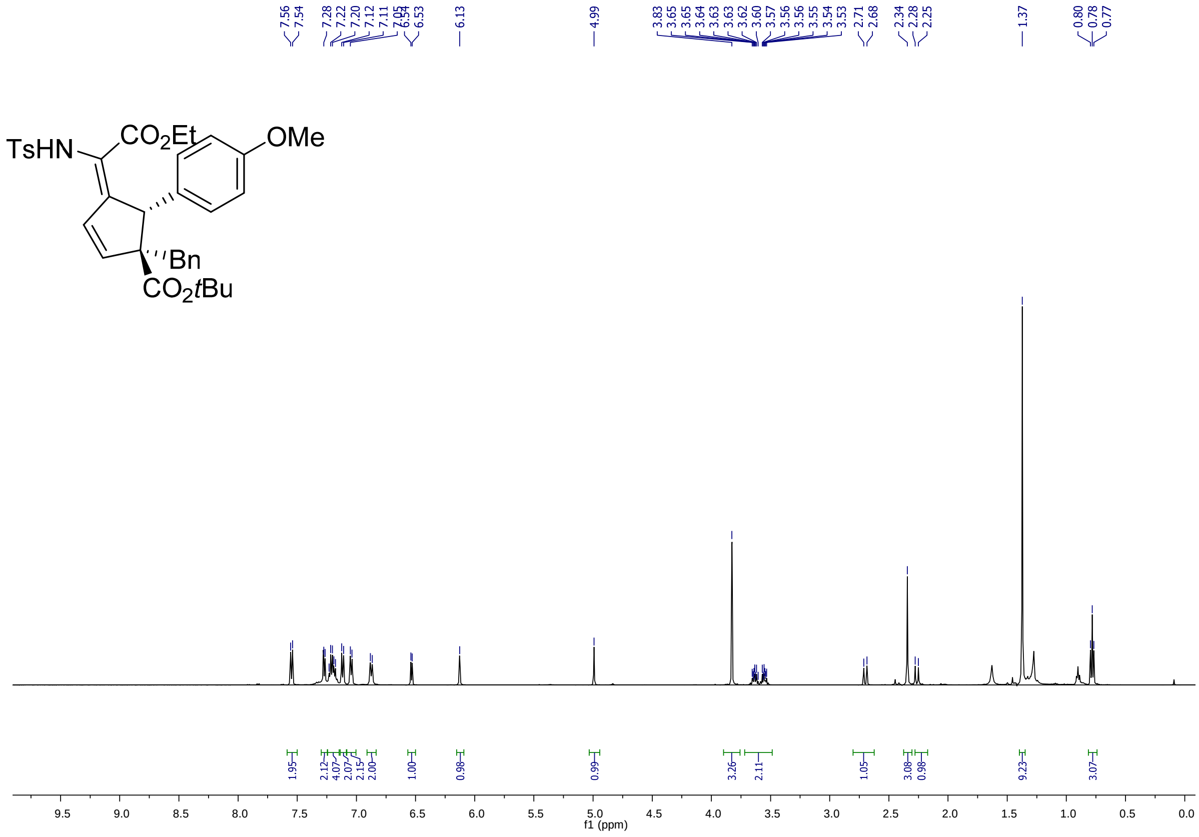
7.56
7.54
7.28
7.22
7.20
7.12
7.11
7.05
6.54
6.53
6.13
4.99
3.83
3.65
3.65
3.64
3.63
3.63
3.62
3.60
3.57
3.56
3.56
3.55
3.54
3.53
2.71
2.68
2.34
2.28
2.25
1.37
0.80
0.78
0.77
TsHN
CO_2Et
OMe
Bn
CO_2tBu
1.95
2.12
4.07
2.07
2.15
2.00
1.00
0.98
0.99
3.26
2.11
1.05
3.08
0.98
9.23
3.07
f1 (ppm)

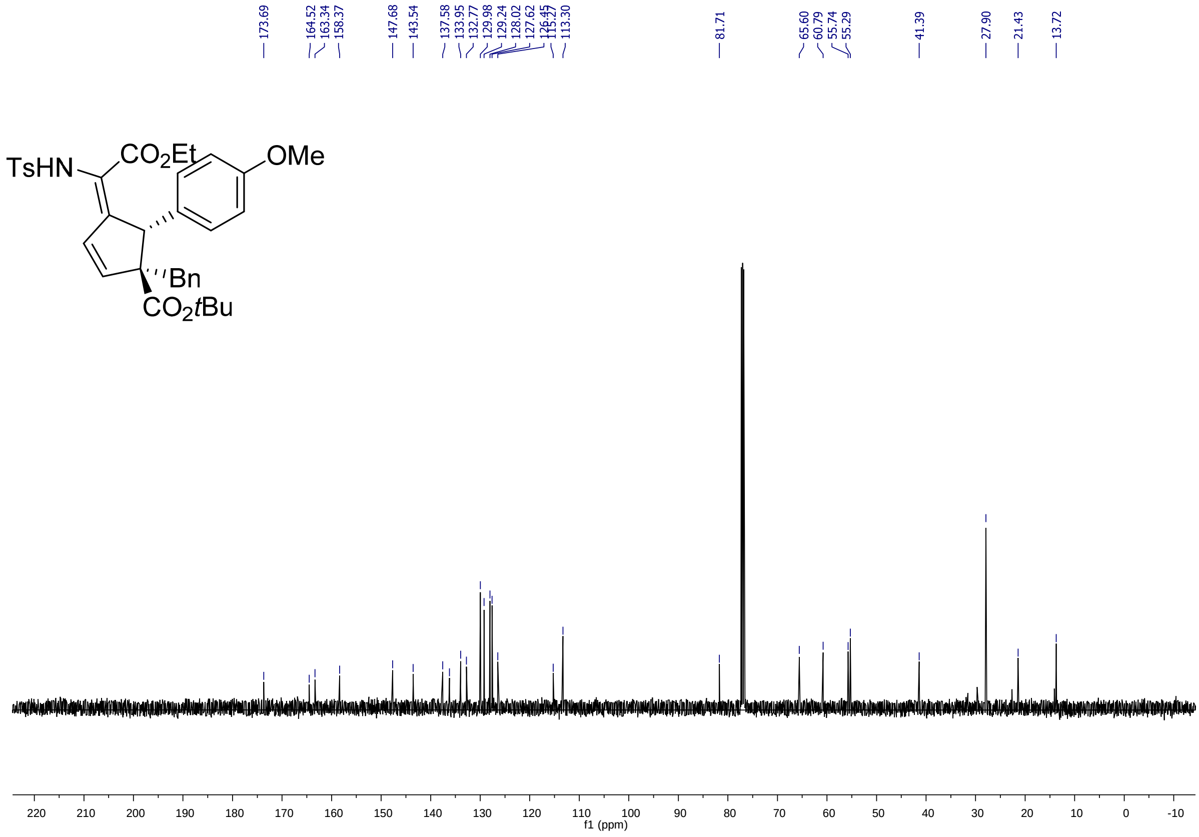
173.69
164.52
163.34
158.37
147.68
143.54
137.58
133.95
132.77
129.98
129.24
128.02
127.62
126.45
115.27
113.30
81.71
65.60
60.79
55.74
55.29
41.39
27.90
21.43
13.72
TsHN
CO_2Et
OMe
Bn
CO_2tBu
f1 (ppm)

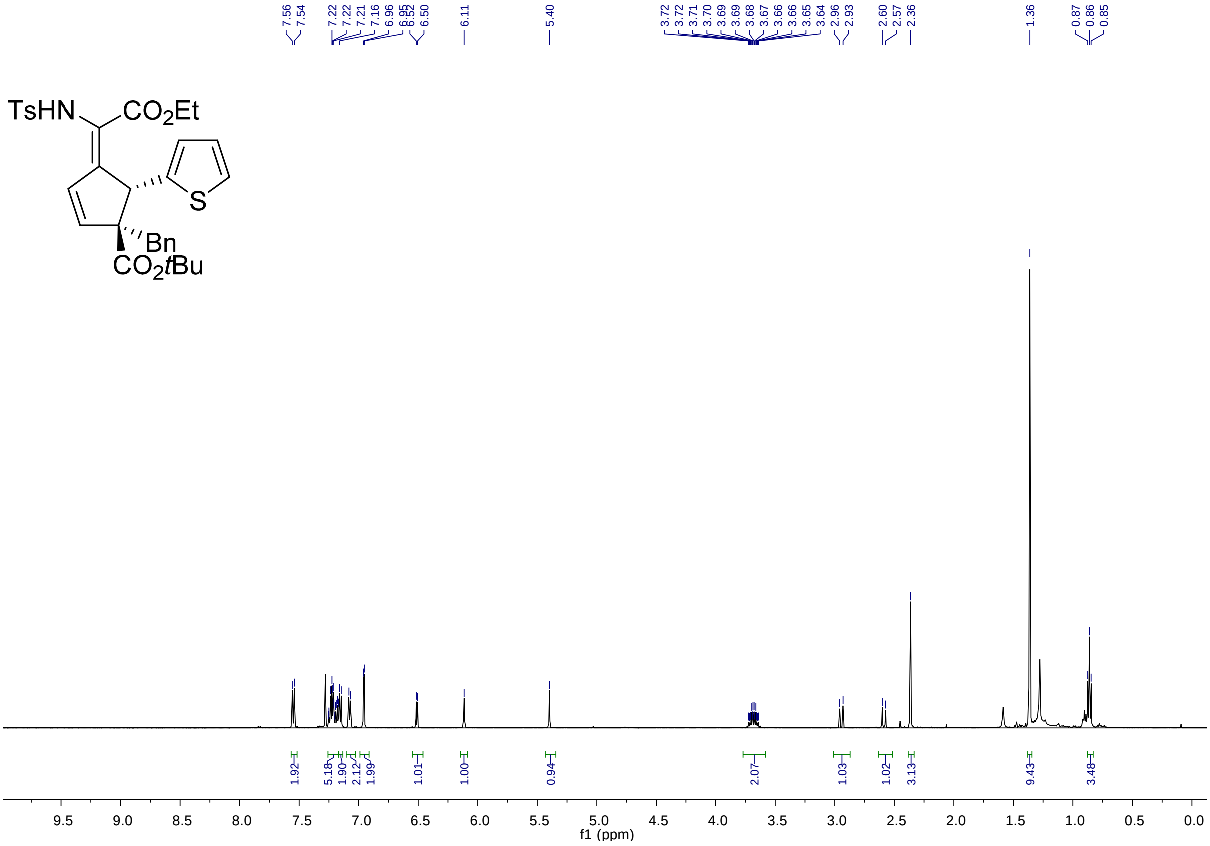

TsHN
CO2Et
S
Bn
CO2tBu
7.56
7.54
7.22
7.22
7.21
7.16
6.96
6.95
6.52
6.50
6.11
5.40
3.72
3.72
3.71
3.70
3.69
3.69
3.68
3.67
3.66
3.66
3.65
3.64
2.96
2.93
2.60
2.57
2.36
1.36
0.87
0.86
0.85
1.92
5.18
1.90
2.12
1.99
1.01
1.00
0.94
2.07
1.03
1.02
3.13
9.43
3.48
9.5 9.0 8.5 8.0 7.5 7.0 6.5 6.0 5.5 5.0 4.5 4.0 3.5 3.0 2.5 2.0 1.5 1.0 0.5 0.0
f1 (ppm)

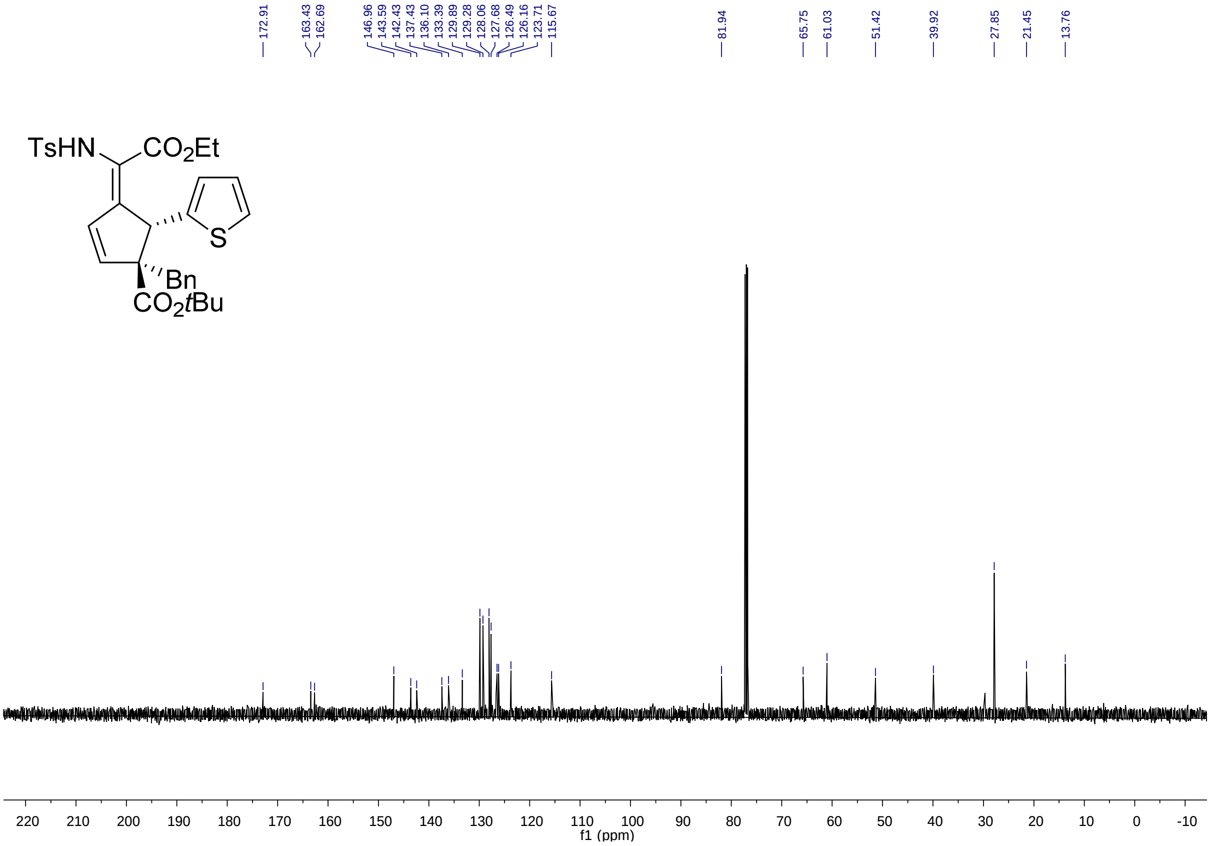

172.91
163.43
162.69
146.96
143.59
142.43
137.43
136.10
133.39
129.89
129.28
128.06
127.68
126.49
126.16
123.71
115.67
81.94
65.75
61.03
51.42
39.92
27.85
21.45
13.76
TsHN
CO2Et
S
Bn
CO2tBu
220 210 200 190 180 170 160 150 140 130 120 110 100 90 80 70 60 50 40 30 20 10 0 -10
f1 (ppm)
